# CLGDS: robust bridge crack detection with YOLO enhanced feature fusion and SIoU optimization

**DOI:** 10.1038/s41598-026-42727-1

**Published:** 2026-04-04

**Authors:** Bao Jiao, Xiao Canjun, Guo Dong, Wang Chenyu, Peng Mi, Zhao Xinping

**Affiliations:** 1 Institute of Mine Intelligence, Chengdu Technological University, Chengdu, 610031 China; 2 China Southwest Architectural Design and Research Institute Corp. Ltd, Chengdu, 610041 Sichuan China

**Keywords:** Engineering, Mathematics and computing

## Abstract

As a key indicator of structural integrity and in-service performance, crack detection is essential for the condition assessment and preventive maintenance of bridges. To address the challenges of detecting cracks with various scales and shapes under low-contrast backgrounds in bridge inspection tasks, this paper proposes a robust detection method named CLGDS. It is based on YOLO11 with enhanced feature fusion and SIoU loss optimization, which effectively improves the accuracy and robustness of crack identification. The proposed framework includes three key innovations. (1) A Cross Stage Partially Large Separable Kernel Attention (C2LSKA) module is integrated in the backbone network to enhanced the representation of crack features in the case of morphologically diverse and complex background interference. (2) A Gathering and Distributing (GD) mechanism serves as the neck network, facilitating multi-scale feature fusion and improving the detection performance for cracks of varying scales and geometrically irregular edges. (3) A Scylla-IoU (SIoU) loss function is introduced to replace the commonly used Complete IoU (CIoU) loss. By explicitly incorporating directional sensitivity and multi-scale adaptability, SIoU effectively mitigates angle-dependent misalignment during bounding box regression. Experimental results demonstrate that CLGDS achieves a mean average precision (mAP@50) of 93.5%, outperforming YOLOv5, YOLOv8, and YOLO11 by margins of +1.5%, +0.6%, and +1.2%, respectively. Furthermore, it attains a mAP@50-95 of 68.5%, significantly higher than that of YOLOv5 (61.3%), YOLOv8 (62.6%), and YOLO11 (65.3%). These results validate the effectiveness of CLGDS in accurate bridge crack detection, providing a solid technical foundation for automated structural health monitoring and preventive maintenance.

## Introduction

As vital lifelines of national transportation infrastructure, the structural health of bridges directly impacts public safety, property security, and societal functioning^1^. The vast number of aging bridges globally necessitates advanced structural health monitoring (SHM) systems, driving extensive research into scientifically grounded assessment and maintenance methodologies. Furthermore, many aging bridges were designed to standards insufficient for current traffic loads, accelerating deck deterioration. In summary, factors such as material degradation, accumulated fatigue, and harsh environmental conditions have led to a surge in various structural damages, including cracking, corrosion, and spalling^1^. Among these, cracks serve as a primary indicator of surface deterioration in bridges^2^. Consequently, their detection and prevention are paramount for effective maintenance. Timely identification and repair can prevent crack propagation, reduce long-term maintenance costs, and ensure traffic continuity and safety. Therefore, accurate crack identification is crucial for enhancing the quality and efficiency of bridge deck inspections.

Early crack detection methodologies traditionally relied on equipment such as bridge inspection vehicles and scaffolding, utilizing instruments like crack gauges, microscopes, and vernier calipers. These approaches depended heavily on manual visual inspection, which was inherently subjective and required substantial expertise and experiential knowledge from inspectors^3^. Research on conventional methods has primarily focused on techniques including wavelet transform^4^, image thresholding^5^, handcrafted feature extraction and classification^6–8^, edge detection-based methods^9^, minimal path-based approaches^10^, among others^11,12^. Although simple and convenient to implement, these methods suffer from significant limitations: high labor intensity, time-consuming procedures, low efficiency, safety risks, high rates of missed defects, and difficulty in inspecting complex bridge structures^13^. Consequently, they often fail to meet the stringent timeliness and accuracy requirements of practical engineering applications.

In response to the limitations of traditional manual inspection, machine learning-based methods have been widely adopted for non-contact crack detection in engineering. By leveraging automated feature learning, these approaches significantly enhance the accuracy and efficiency of bridge crack inspection. Crack detection approaches can be broadly categorized into supervised and unsupervised methods, depending on their reliance on labeled data. Supervised learning utilizes annotated training data to establish pixel-level mappings between input images and crack locations, offering high accuracy and computational efficiency^14,15^. However, its performance relies heavily on the availability of labeled data. In contrast, unsupervised methods identify inherent data patterns without requiring labels, thereby eliminating manual annotation costs at the expense of quantitative assessment capacity^16,17^. Compared to earlier approaches, machine learning-based methods exhibit greater robustness, but their detection accuracy often drops significantly in real-world scenarios.

**Fig. 1:**
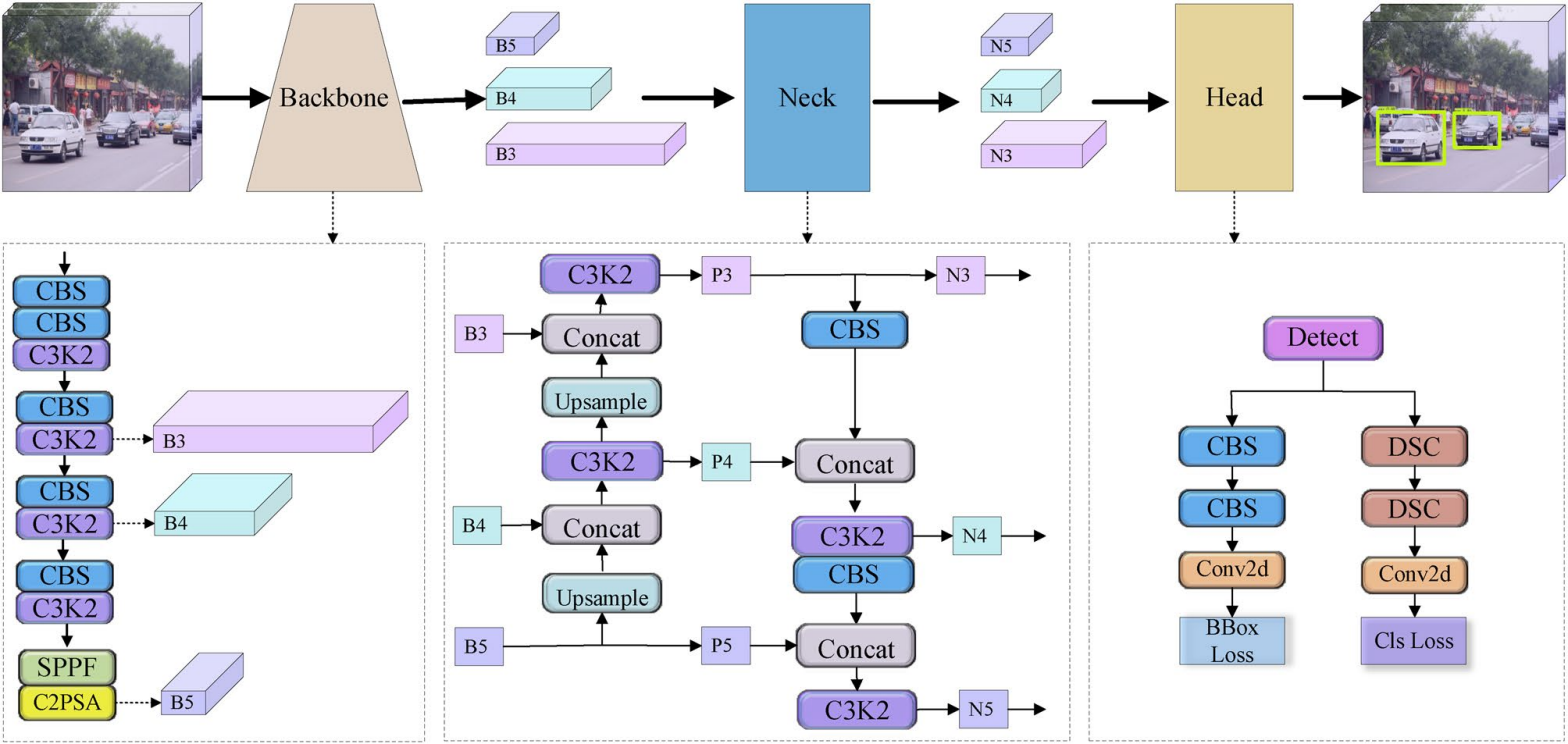
YOLO11 network architecture.

With advancements in computer hardware, the integration of high-resolution optical imaging with computer vision techniques has become the mainstream approach for structural defect acquisition and intelligent detection. Deep learning, a cornerstone of modern computer vision, excels in image processing by automatically extracting complex crack features without manual intervention^3^, and thus has become a predominant research focus in this field. While direct image-based crack detection is a primary application, the effectiveness of deep learning models is fundamentally constrained by the scarcity and variability of high-quality labeled data in field environments. To enhance model robustness and generalizability, significant research has focused on designing specialized Deep Convolutional Neural Network (DCNN) architectures and integrating them with sophisticated data augmentation strategies. This approach often extends to multimodal data, enriching the feature learning process critical for accurate damage diagnosis, including crack-related degradation. The evolution of these methods is evident in recent literature. An early application used a deep CNN to analyze augmented modal experimental data, directly linking connection stiffness reduction in steel bridges to measurable dynamic signatures^18^. Subsequent work integrated an artificial neural network with a hybrid stochastic optimization algorithm to improve the precision of damage parameter identification under uncertainty, a method applicable to quantifying crack-induced structural changes^19^. More recently, research has demonstrated the power of hybrid sequential models. For instance, a 1DCNN-BiLSTM network, combined with data augmentation, was developed to detect stiffness reduction in truss bridges from vibration signals, capturing spatiotemporal patterns indicative of incipient damage^20^. Similarly, a two-step framework employing a Convolutional LSTM (ConvLSTM) on augmented time-series data was proposed to identify and track damage progression in bridge structures, effectively modeling its temporal evolution^21^. Collectively, these DCNN-centric approaches, fortified by data augmentation, serve as advanced feature extractors. They transform raw or augmented sensor data—whether vibrational, modal, or temporal—into robust, high-dimensional representations of structural integrity.

Based on the architectural paradigms, deep learning-based detectors are categorized into two main types: single-stage and two-stage detectors^3,22^. The robust feature maps generated by these DCNN modules serve as the input for the subsequent detection heads in both paradigms. This enables precise crack localization and classification within a unified deep learning framework. Two-stage detectors, such as the R-CNN^23^, Fast R-CNN^24^, and Faster R-CNN^25^ series, first generate region proposals and then perform classification and bounding box regression. In contrast, single-stage detectors like You Only Look Once (YOLO)^26,27^ and Single Shot MultiBox Detector (SSD)^28^ directly predict bounding boxes and class probabilities from input images, eliminating the region proposal step. Comparatively, two-stage methods achieve higher detection accuracy at the cost of greater computational complexity, while single-stage approaches offer faster inference speeds with moderately reduced accuracy.

**Fig. 2:**
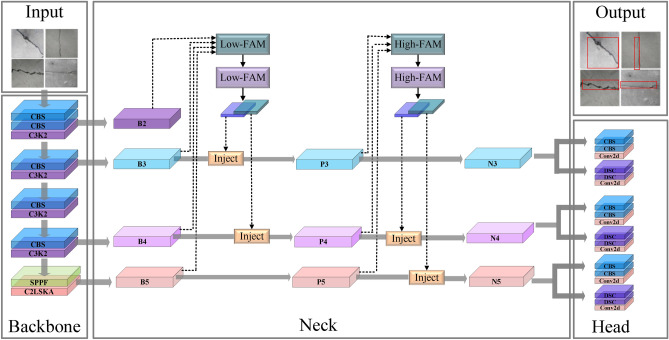
The proposed CLGDS network architecture.

Deep learning-based single-stage detectors have revolutionized object detection, enabling significant improvements in accuracy, speed, and computational efficiency through end-to-end architectural innovation. As a result, they have become the mainstream approach in the field. Recent studies indicate that these end-to-end single-stage methods, particularly the YOLO series, have replaced earlier machine learning techniques as a mainstream solution for bridge crack detection^29^. Research on YOLO algorithms focuses on two main optimization strategies: architectural modifications to improve accuracy^30–32^, and model compression techniques to enhance inference speed^33–35^. While YOLO-series detectors generally deliver high detection accuracy and speed, their performance drops significantly in bridge crack detection due to challenges such as the slender shape of cracks, low contrast with the background, and various interfering factors. Therefore, detecting bridge cracks in real-world scenarios remains a challenging task.

This work proposes CLGDS, an enhanced YOLO11 network for robust bridge crack detection. While the C2PSA module in YOLO11 improves multi-scale feature extraction, its ability to capture fine crack details remains limited^36^. In contrast, the LSKA module shows strong adaptability to varying kernel sizes^37^. To combine these complementary strengths, we propose a novel C2LSKA module, a heterogeneous perception module that integrates C2PSA with LSKA to detect cracks across multiple scales. To further sharpen crack edges and suppress background interference, we introduce the Gathering and Distributing (GD) mechanism as a feature fusion block^38^. This mechanism integrates encoder-decoder features at corresponding scales through multi-stage fusion. Given the geometric complexity of cracks and the superiority of SIoU in modeling such relationships, we adopt SIoU as the regression loss function. In brief, the proposed CLGDS network aims to capture fine-grained features, fuse local details with global semantics, and model crack geometry. The key improvements include:

1. C2LSKA Module: The core design of C2LSKA is to enhance feature discriminability by capturing multi-dimensional representations through efficient computation and interaction. By decomposing large-kernel spatial attention into a series of separable small-kernel operations^37^, C2LSKA effectively captures salient spatial information, thereby improving detection performance. This design strikes a balance between accuracy and computational efficiency, rendering C2LSKA well-suited for dense prediction in resource-constrained settings.
2. GD Mechanism: We replace YOLO11’s original up-sampling and down-sampling modules with the GD mechanism^38^. This GD architecture integrates local and global contextual representations to enhance crack edge delineation and suppress background noise. By hierarchically aggregating features within global receptive fields and redistributing them across scales, GD establishes efficient cross-level interactions. This significantly improves multi-scale fusion while optimizing accuracy-latency tradeoffs across model sizes.
3. SIoU Loss: We employ the SIoU loss^39^ to optimize bounding box regression and enhance localization precision. SIoU provides a comprehensive matching metric by jointly evaluating positional offset, scale discrepancy, and angular alignment between predicted and ground truth (GT) boxes. Its aspect ratio constraint term actively reduces shape variance by penalizing significant deviations, thus enforcing geometric consistency. Furthermore, SIoU speeds up convergence and enhances matching precision through an angular penalty that considers the orientation between predicted and GT boxes.

**Fig. 3:**
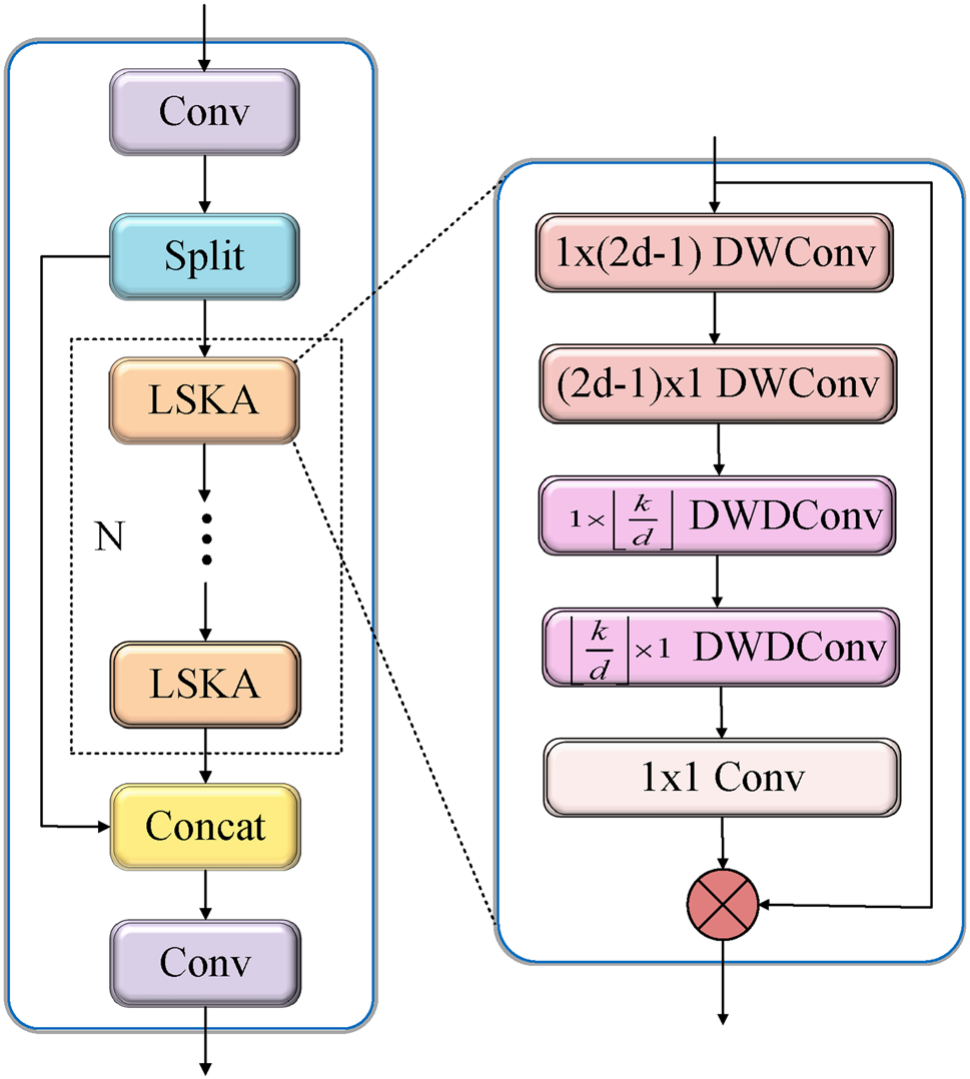
The proposed C2LSKA module.

The paper is organized as follows: “Related works” reviews related works and discusses their merits and limitations. “Methodology” introduces the proposed methodology. “Experiments” and “Experiment and result analysis ” describe experiments and results. “Discussion” and “Conclusions” offer discussion and conclusions.

## Related works

### YOLO for object detection

Bridge crack detection is critical for structural maintenance and represents a canonical object detection task. As representative single-stage detectors, both SSD^28^ and YOLO^26,27^ predict bounding boxes directly from convolutional feature maps, differing primarily in their architectural design and feature fusion strategies. A key innovation of the YOLO series is its formulation of detection as a spatially discrete grid prediction problem. These methods achieve a favorable trade-off between accuracy and real-time performance, which has led to their widespread adoption in computer vision. Since its inception in 2016, the YOLO series has evolved substantially. Its development spans three phases: foundational (v1–v3), performance optimization (v4–v7), and architectural innovation (v8–the present). To further improve the speed–accuracy trade-off, continuous optimizations have been made to the core YOLO architecture. Key enhancements focus on three aspects: backbone network design, cross-layer feature fusion mechanisms, and loss functions^40^.

The backbone network is the core feature extractor in object detection, significantly influencing both accuracy and inference speed. It uses custom convolutional blocks to build deep architectures, achieving a favorable speed-accuracy trade-off^41^. For instance, the Cross Stage Partial Network (CSPNet) significantly expands the receptive field of DarkNet-53 through cross-stage partial connections and spatial pyramid pooling (SPP)^42^. By partitioning feature maps, CSPNet improves gradient flow integration, reducing computational redundancy and improving learning efficiency. To enable lightweight deployment, models like YOLO-Mobile^43^ and YOLO-Nano^44^ replace standard convolutions with separable or pointwise convolutions, greatly reducing computational cost (FLOPs). Furthermore, integrating attention mechanisms^45^ and transformers^46^ also boosts recall for small objects and improves detection robustness under occlusion.

Feature interaction mechanisms address key challenges in object detection, such as scale variations, occlusions and small targets. They optimize how information is integrated across hierarchical levels, spatial locations and semantic contexts. Multi-scale feature fusion methods have evolved from the initial Feature Pyramid Network (FPN)^41^ to current approaches like PANet^47^ and BiFPN^48^, progressively enhancing cross-scale interactions. Meanwhile, spatial-channel attention mechanisms work synergistically to improve feature representation^45,49^. Task-decoupling strategies further mitigate conflicts between classification and regression by promoting task-specific feature learning^50^. In recent research, the GD mechanism in GOLD-YOLO is designed with a novel cross-level fusion architecture to overcome the limitations of nearest-neighbor upsampling^38^, demonstrating outstanding detection performance.

**Fig. 4:**
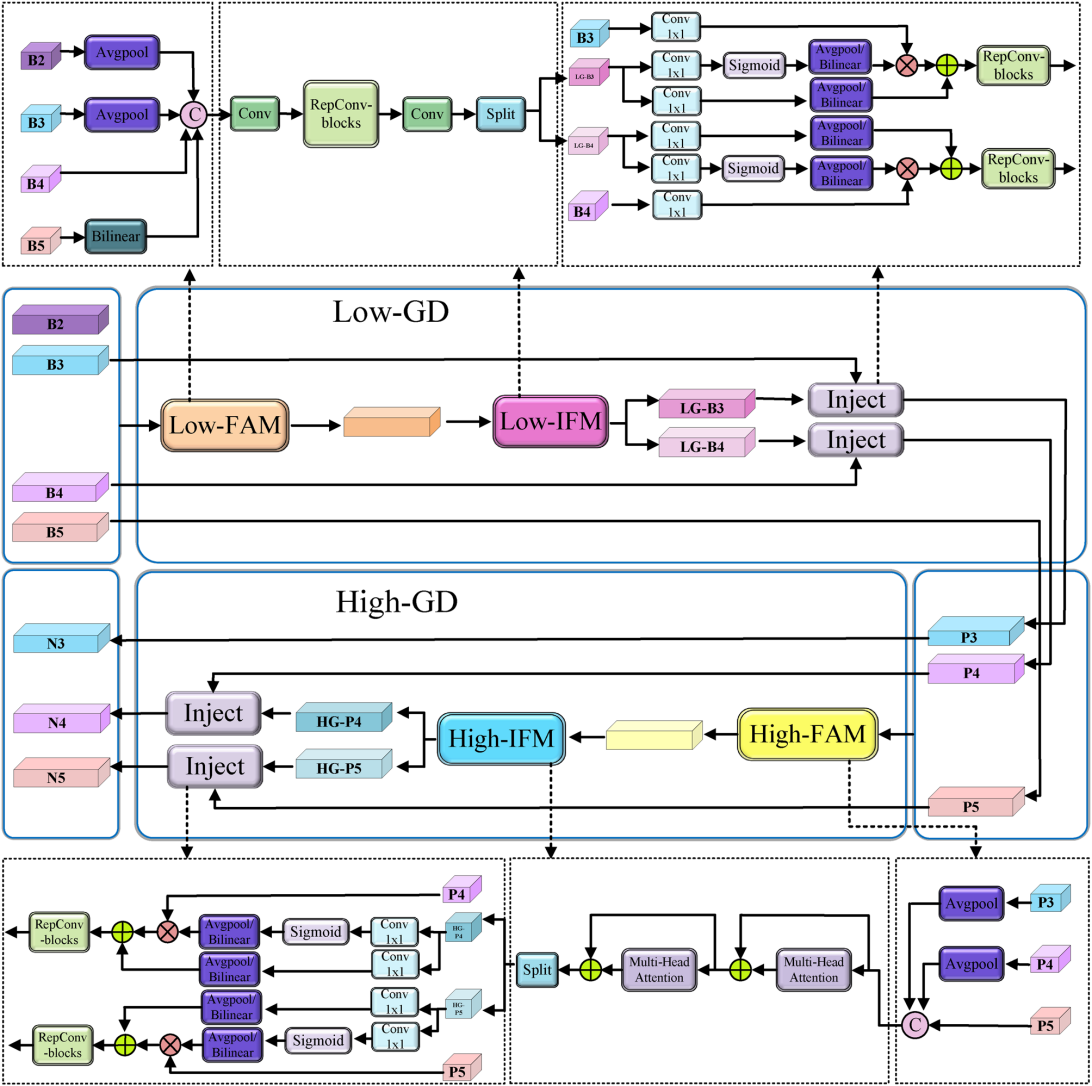
The GD mechanism.

The loss function defines the optimization objective in object detection, directly governing localization accuracy and classification reliability. It requires the joint optimization of two tasks: bounding box regression and object classification. The YOLO algorithm unifies these into a single regression framework with three loss components: localization loss, confidence loss, and classification loss. The localization loss has evolved from Mean Squared Error (MSE) to more geometry-aware IoU-based losses^26,27^. For classification, early YOLO versions used cross-entropy loss, which struggled with hard examples. This was addressed by Focal Loss for class imbalance, and later refined by Varifocal Loss (VFL) for better weighting of positive and negative samples^51^. In short, YOLO’s loss has evolved from fixed formulations to adaptive variants.

In summary, continuous improvements to the backbone, neck, and loss functions have enabled the YOLO series to achieve strong generalization performance in general object detection. However, existing methods still struggle with bridge crack detection, where factors such as target slenderness, edge ambiguity, shape diversity, and complex backgrounds significantly limit their performance. Inspired by the aforementioned research, this paper proposes CLGDS, an enhanced algorithm for bridge surface crack detection. Its core innovations include: (1) the C2LSKA module for better feature extraction, (2) the GD mechanism for richer feature representation, and (3) the SIoU loss to enhance prediction accuracy.

### YOLO11 network architecture

The YOLO series is the widely adopted benchmark in object detection, undergoing continuous refinement driven by the demand for real-time performance. Based on YOLOv8^22^, YOLO11 retains the single-stage detection paradigm while enhancing performance and flexibility through architectural and training optimizations. It provides five scaled variants (11n/s/m/l/x) that balance model size, computation, and speed. Architecturally, YOLO11 maintains the backbone-neck-head design, ensuring robust detection while supporting tasks like instance segmentation. Given its competitive accuracy, YOLO11 is selected as the baseline for our bridge crack detection research.

**Fig. 5:**
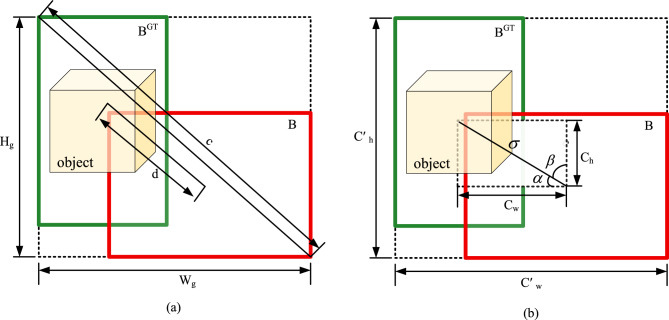
The CIoU and SIoU loss functions. (**a**) CIoU, and (**b**) SIoU.

Building upon YOLOv8, YOLO11 introduces significant architectural innovations. For feature fusion, it adopts enhanced strategies that integrate multi-scale information more effectively, such as the improved Path Aggregation Network (PANet) and optimized feature pyramid structures, enabling the model to capture finer object details. In label assignment and alignment, it incorporates refined techniques like SimOTA and the Task-Aligned One-Stage Object Detection (TOOD) head, which improve both classification and localization accuracy by better aligning predicted boxes with ground truths. Furthermore, YOLO11 benefits from advanced training strategies, including self-supervised pre-training and knowledge distillation, collectively contributing to its state-of-the-art performance.

YOLO11 employs a classic backbone-neck-head architecture for end-to-end optimization, seamlessly progressing from feature extraction to final object detection and classification. This overall pipeline is illustrated in Figure 1.

YOLO11’s backbone introduces a highly reconfigurable design with two key innovations for better multi-scale representation and efficiency. (1) C3K2 module: It replaces the standard C2f block with a dynamic branch mechanism. This improves feature extraction flexibility, maintains parameter efficiency, and widens the receptive field. (2) PSA mechanism^52^: It is integrated within the SPPF layer^53^, using multi-head attention to capture long-range spatial dependencies. The neck network is positioned between the backbone and detection heads. Based on a Feature Pyramid Network (FPN), it fuses multi-scale features for semantic enhancement. YOLO11 further incorporates a C3K2 module here, which aggregates cross-level features and restructures propagation paths, which avoids task conflict. It also employs dynamic label assignment and position-aware loss to better match training samples across scales. Even with a lightweight design, the head effectively combines multi-scale features from the backbone and neck. This synergy improves both classification and localization, leading to stronger generalization in complex scenes.

## Methodology

This section presents the overall architecture of the proposed CLGDS, as shown in Fig. 2. The network consists of three core components: (1) a backbone enhanced with a C2LSKA module for richer feature extraction; (2) a neck using a GD mechanism for multi-scale feature fusion; and (3) a detection head optimized with the SIoU loss function. In this framework, the backbone extracts hierarchical features, the neck fuses them across scales, and the head outputs the final crack localization.

### C2LSKA module

Compared with traditional attention mechanisms, the C2PSA module in the YOLO11 backbone improves focus on complex, occluded objects through multi-scale convolution and adaptive channel weighting. However, its effectiveness diminishes when detecting bridge cracks, which vary greatly in shape, size, and orientation. For thin, low-contrast cracks, C2PSA may lose critical details, lowering accuracy. It also struggles to adapt to complex crack morphologies and positional shifts, affecting precise center and boundary localization. Furthermore, noise from uneven lighting, shadows, and surface textures during image acquisition can further reduce the model’s detection accuracy and stability.

**Fig. 6:**
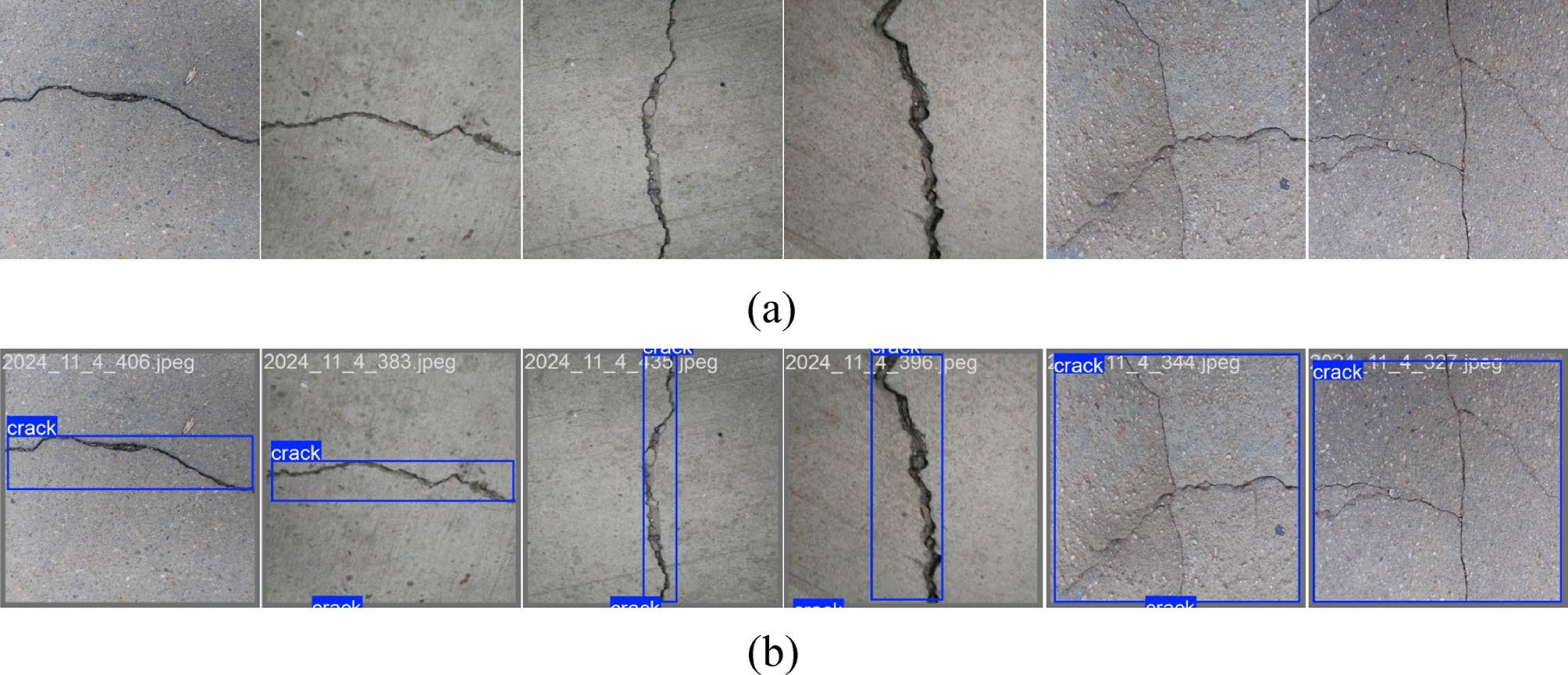
Samples from the crack dataset. (**a**) original images, and (**b**) corresponding labels.

To address the aforementioned issues, we propose the C2LSKA module to enhance the YOLO11 backbone, as shown in Fig. 3. Its core innovation lies in replacing the PSA module in C2PSA with N serial LSKA blocks^37^, which decompose standard 2D convolution into cascaded horizontal and vertical 1D kernels. This design greatly lowers computational cost while preserving a large receptive field, improving the modeling of long-range dependencies. The module also effectively suppresses noise, increasing robustness against uneven lighting and complex textures.

As illustrated in Fig. 3, processing begins with the feature map from the previous layer, denoted as F, as follows. After an initial convolution yielding $F^{C}$, the features are split. One path $F^{C}_{0}$ flows through a series of N LSKA blocks for enhanced processing, resulting in $F^{L}_{N}$. This is then merged with the other path $F^{C}_{1}$ to form $F^{\prime }$. A final convolution integrates these combined features, balancing detail preservation with efficient computation for the subsequent network stages.

The sub-map $F^{C}_{0}$ (size H $\times$ W) is processed through N serially connected LSKA modules. In the first module, $F^{C}_{0}$ is simultaneously convolved with two depthwise separable kernels: a horizontal 1$\times$(2d-1) and a vertical (2d-1)$\times$1 kernel. This produces the output feature $\bar{Z}^{C}_{1}$ (see formula 1), representing the 1D depthwise separable convolution. The operator $*$ denotes convolution.

1 $$\begin{aligned} \bar{Z}^{C}_{1}=\sum _{H,W}W^{C}_{(2d-1)\times 1}*(\sum _{H,W}W^{C}_{(2d-1)\times 1}*F^{C}_{0}) \end{aligned}$$

Next, the feature maps $\bar{Z}^{C}_{1}$ are separately convolved with two 1D depthwise separable dilated kernels: a horizontal and a vertical kernel. This produces the output $Z^{C}_{1}$ (see formula 2) from the one-dimensional depthwise separable dilated convolution. Here, *k* is the maximum receptive field size and *d* is the dilation rate.

2 $$\begin{aligned} Z^{C}_{1}=\sum _{H,W}W^{C}_{ \lfloor \frac{k}{d} \rfloor \times 1}*(\sum _{H,W}W^{C}_{1 \times \lfloor \frac{k}{d} \rfloor }*\bar{Z}^{C}_{1}) \end{aligned}$$

**Fig. 7:**
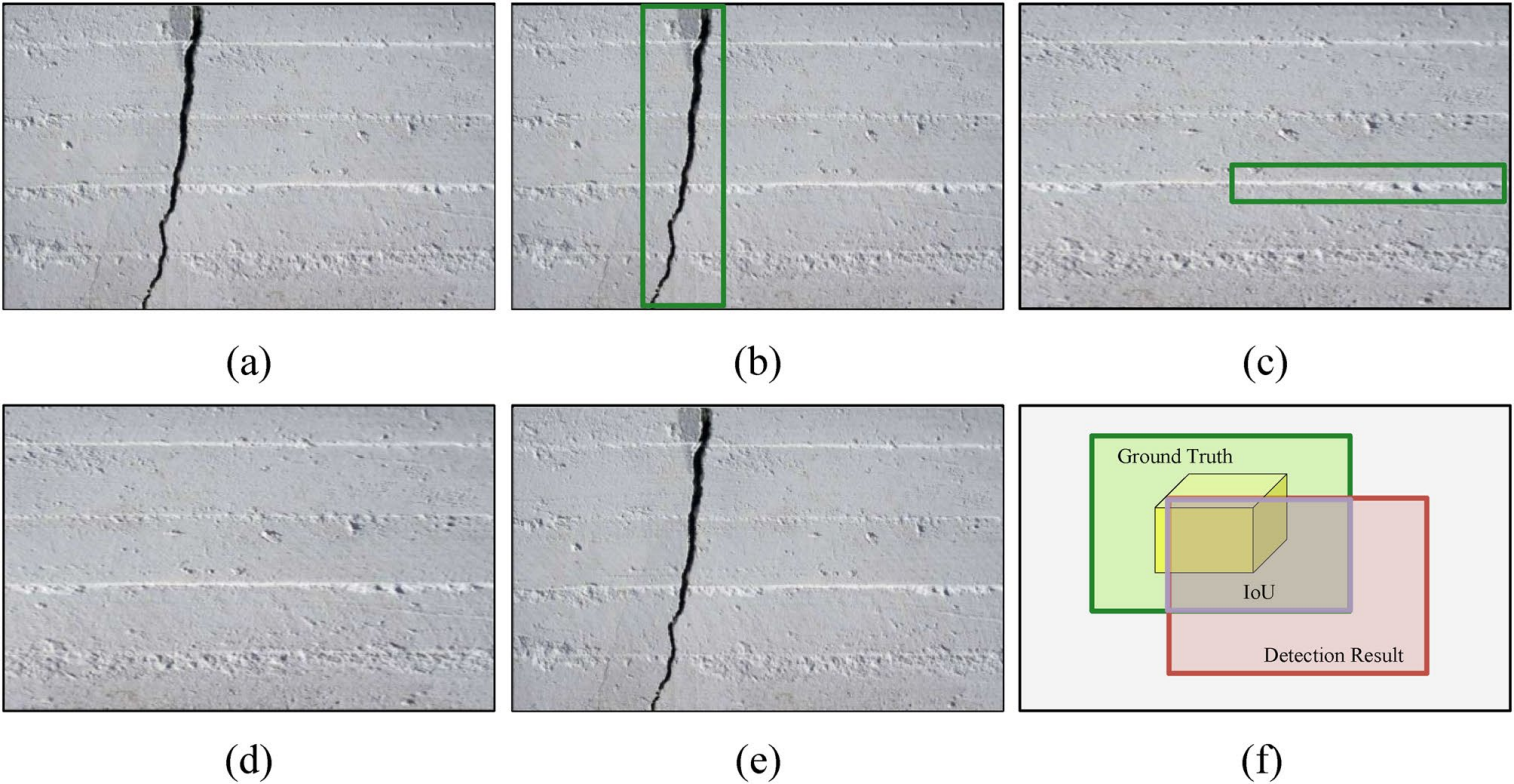
Schematic diagram of key evaluation metrics for crack detection. (**a**) Example crack image, (**b**) TP: correct crack detection, (**c**) FP: false alarm, (**d**) TN: correct rejection, (**e**) FN: missed crack, and (**f**) intersection over union (IoU) visualization.

Finally, $Z^{C}_{1}$ is transformed by a 1$\times$1 convolution into an attention map $A^{C}_{1}$. This map is then element-wise multiplied with $F^{C}_{1}$ to produce the module’s final output $F^{L}_{1}$ (see formula 3). Feature $F^{C}_{0}$ is processed through N serially connected LSKA modules, yielding the output feature map $F^{L}_{N}$.

3 $$\begin{aligned} F^{L}_{1}=(W_{1\times 1}*Z^{C}_{1} ) \otimes F^{C}_{1} \end{aligned}$$

**Fig. 8:**
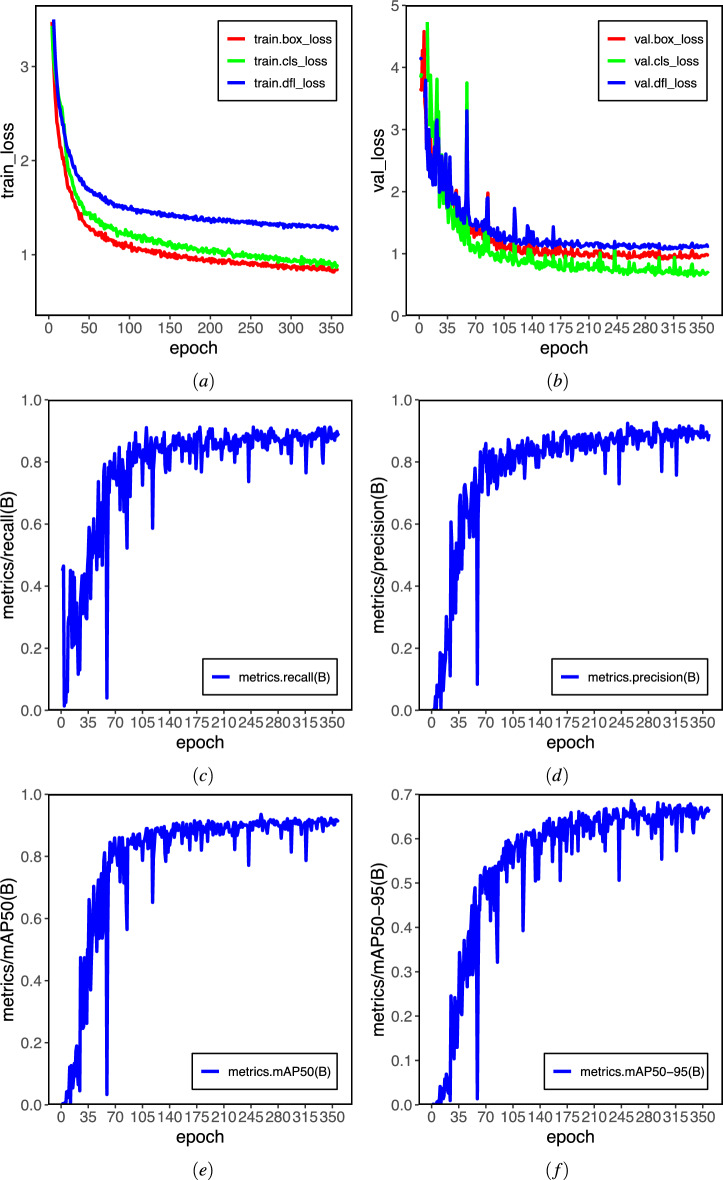
Training curves for loss functions and performance metrics. (**a,b**) Box, classification (cls), and distribution focal (dfl) loss curves on the training and validation sets, respectively, (**c**) recall, (**d**) precision, (**e**) mAP@50, and (**f**) mAP@50-95.

The performance of our proposed C2LSKA module is critically dependent on several core hyperparameters, which collectively govern its multi-scale receptive field and representational capacity for crack feature learning. The primary configurable parameters include: the number of sequentially stacked LSKA units (denoted as N), the base kernel size for large-kernel depthwise separable convolutions (denoted as k), and the dilation rate applied within these convolutions (denoted as d). Specifically, N controls the depth of the sequential attention refinement pathway. k and d together define the effective receptive field of the depthwise separable convolutions. The synergistic tuning of (N, k, d) directly governs the trade-off between detection accuracy–especially for micro-cracks and under noisy conditions—and computational efficiency. A detailed discussion on the selection and impact of these parameter values will be provided in the ablation study that follows.

### GD mechanism

YOLO11 uses an improved cross-stage feature fusion strategy based on PANet to combine information from different levels. However, this design requires information to pass through multiple intermediate layers when moving between non-adjacent levels. This multi-step process causes two main problems: (1) fine details of small cracks are gradually lost as features move to deeper layers, and (2) large-scale crack features lack sufficient semantic information when moving to shallower layers. In bridge crack detection, these losses of information reduce accuracy across scales. Additionally, because the neck network does not fully use details from shallow layers, detecting small cracks becomes harder, leading to more missed detections.

**Fig. 9:**
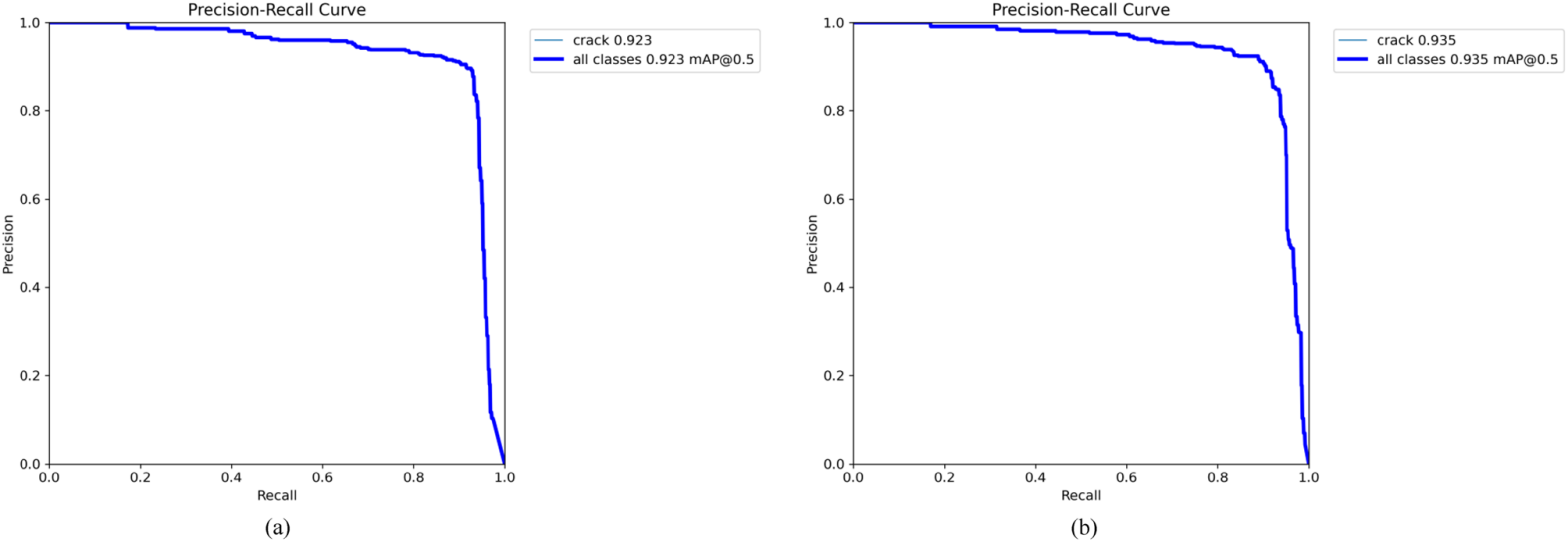
Precision-recall curves for performance evaluation. (**a**) YOLO11, and (**b**) the proposed algorithm.

To address the feature degradation caused by recursive fusion, we integrate the GD mechanism^38^ into YOLO11’s neck network. This mechanism leverages a combination of convolution and self-attention to achieve global fusion of cross-layer features, then efficiently redistributes this fused global context back to all feature levels. This design preserves multi-scale information effectively, notably retaining fine-grained shallow features, which significantly enhances the neck network’s fusion capability.

As shown in Fig. 4, the GD mechanism uses a gather-and-distribute workflow for efficient feature fusion. Its architecture consists of three parts: a lower-stage branch (Low-GD), a higher-stage branch (High-GD), and an information injection (Inject) module. The architecture employs a dual-branch design: the Low-GD branch processes larger feature maps for large-object detection, while the High-GD branch handles smaller ones to enhance small-object recognition. The Inject module then optimizes both branches by distributing aggregated global information across levels via a lightweight attention mechanism. Internally, each GD branch contains a Feature Alignment Module (FAM) for spatial and cross-scale alignment, and an Information Fusion Module (IFM) for effective fusion of the aligned features.

As illustrated in Figs. 3 and  4, the feature pyramid (B2, B3, B4, B5) from the backbone is sequentially processed by the GD mechanism. In the Low-GD stage, the Low-FAM module first aligns backbone features (B2-B5) via interpolation and pooling. The Low-IFM then extracts global features, outputting LG-B3 and LG-B4. The Injection following the Low-GD module distributes and injects LG-B3 and LG-B4 into features B3 and B4 respectively, producing the globally fused cross-layer features P3 and P4. Feature B5 passes through directly as P5, forming the intermediate pyramid (P3, P4, P5). In the High-GD stage, the High-FAM aligns the (P3–P5) pyramid via pooling. The High-IFM extracts global features via multi-head attention, outputting HG-P4 and HG-P5. Similarly, the Injection following the High-GD module injects HG-P4 and HG-P5 into features P4 and P5 respectively, obtaining globally fused multi-scale features N4 and N5. Feature P3 passes through as N3, completing the final pyramid (N3, N4, N5), which is sent to the detection head.

### SIOU loss function

The choice of loss function for bounding box regression directly impacts detection performance. YOLO11 uses Binary Cross-Entropy (BCE) for classification, and combines Distribution Focal Loss (DFL) with CIoU for regression^36^. CIoU considers area, center distance, and aspect ratio. However, for crack detection, CIoU struggles with large variations in object size and shape, and fails to balance easy versus hard samples effectively. To address these limitations, we adopt the SIoU loss^39^, which integrates angle, distance, shape, and IoU geometric constraints to dynamically weight the loss. By explicitly modeling angular and shape priors, SIoU provides robust, geometry-aware regression suitable for complex detection scenarios.

The SIoU loss outperforms CIoU primarily due to two key mechanisms. First, its center distance constraint with dynamic gradient adjustment uses the aspect ratio of a minimum bounding rectangle to constrain box distance. This dynamically reduces the gradient impact from high-confidence samples (e.g., clear horizontal cracks), refining detection accuracy. Second, its angular sensitivity corrects rotational deviations by sensing angular changes, which is crucial for detecting features like diagonal cracks. These innovations overcome CIoU’s reliance on static metrics and accelerate model convergence by combining dynamic gradient modulation with geometric constraints, ultimately enhancing both the accuracy and speed of crack detection. The computational workflows for both loss functions are shown in Fig. 5.

**Fig. 10:**
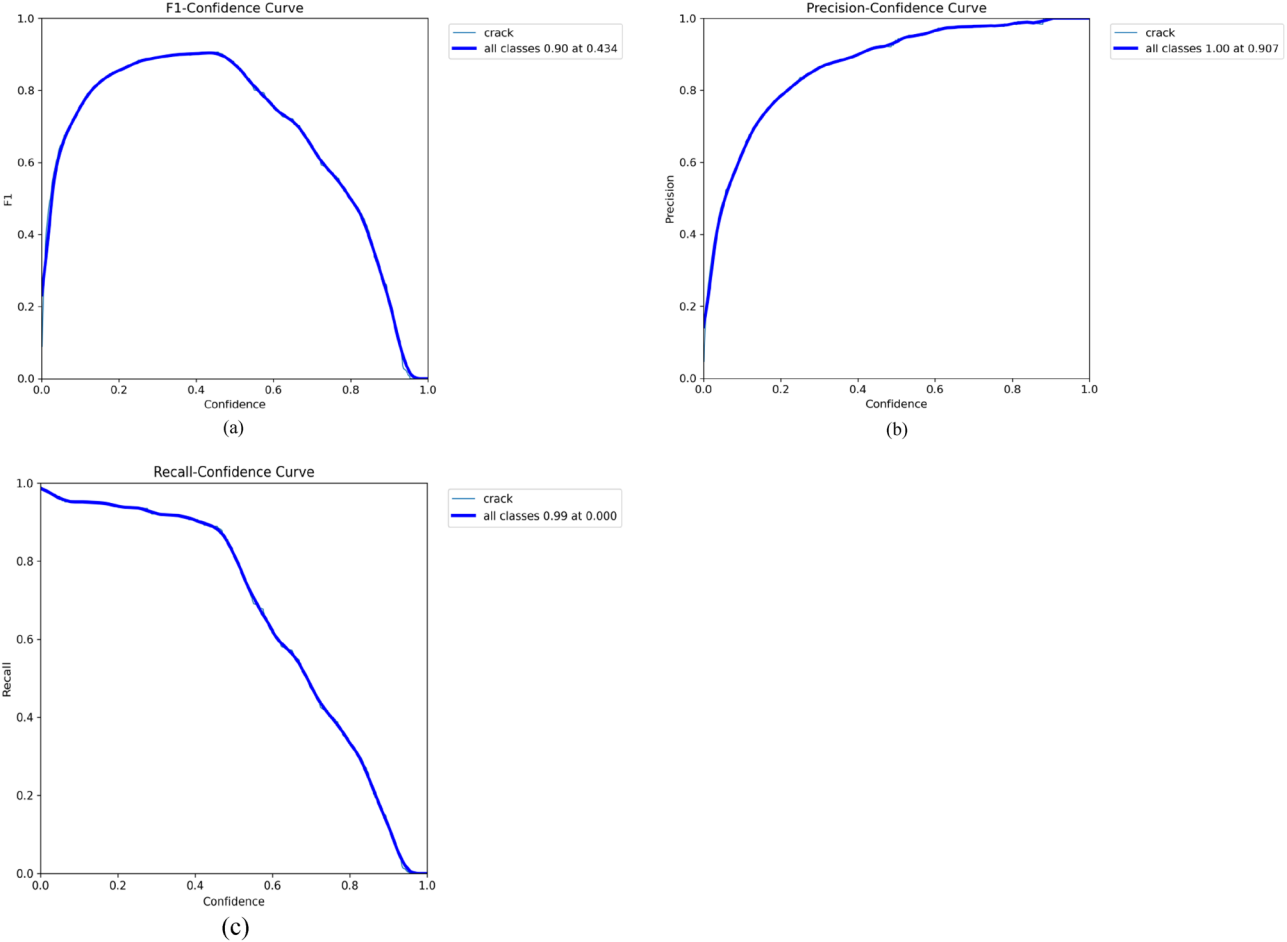
Performance diagnostic curves. (**a**) F1-confidence, (**b**) precision-confidence, and (**c**) recall-confidence.

As depicted in Fig. 5, the predicted and GT boxes for a crack are denoted as B and $B^{GT}$, respectively. Their minimum enclosing rectangle has width $W_{g}$ and height $H_{g}$. The center distance between the two boxes is d, and the diagonal length of their smallest enclosing region is c. The SIoU loss function is then given by Eq.  (4)^39^:

4 $$\begin{aligned} L_{SIoU}=1-IoU+\frac{\Delta + \Omega }{2} \end{aligned}$$

where $\Lambda$ denotes the angle cost, $\Delta$ represents the distance cost, $\Omega$ corresponds to the shape cost, and IoU indicates the intersection over union^39^. Note that the computation of both $\Delta$ and $\Omega$ depends on the prior calculation of $\Lambda$.

The objective function of the proposed method is defined as follows (see formula 5):

5 $$\begin{aligned} L_{Total}=\lambda _{1} L_{BCE}+\lambda _{2}L_{DFL}+\lambda _{3}L_{SIoU} \end{aligned}$$

where $\lambda _{1}$, $\lambda _{2}$, and $\lambda _{3}$ are hyperparameters, and $L_{BCE}$, $L_{DFL}$, and $L_{SIoU}$ are the loss functions of BCE, DFL, and SIoU, respectively.

Following our improvement plan, we developed the enhanced YOLO11 model (as shown in Fig. 2). During detection, its backbone network uses a C2LSKA module to improve feature extraction in complex backgrounds. The neck then employs a GD mechanism to fuse multi-scale features for richer representations. Finally, the detection head applies an SIoU loss for bounding box regression, which jointly optimizes angle, distance, shape, and IoU to robustly identify cracks despite large variations in size and shape.

## Experiments

This section first presents the high-resolution bridge surface image dataset used for training and testing. It then details the implementation of the crack detection network, and finally introduces the evaluation metrics.

### Datasets

After determining the algorithm structure, a substantial number of appropriate crack images were collected to form the training and test sets. The data provided in this paper are sourced from the Baidu Paddle AI Studio platform (https://aistudio.baidu.com). The original dataset contains a total of 1,424 bridge appearance images, each with dimensions of 1024$\times$1024 pixels, as illustrated in Fig. 6a. Since some original images contained redundant objects or highly similar backgrounds, we prioritized the selection of images.

We used Easy Deep Learning (EasyDL) to annotate the crack types and locations. These annotated images were then used for model training and prediction. The labeled image examples in Fig. 6b are from a dataset that was split 7:3 for training and testing and will be made publicly available.

### Experimental setup

Prior to training, the experimental parameters and software environment were configured as follows. All experiments were performed on a workstation running Ubuntu 22.04, equipped with an Intel Core i9-14900KF CPU and an NVIDIA GeForce RTX 5070 Ti GPU (16 GB VRAM). The deep learning framework was built with Python 3.10.19 and PyTorch 2.9.0 within the PyCharm IDE.

**Fig. 11:**
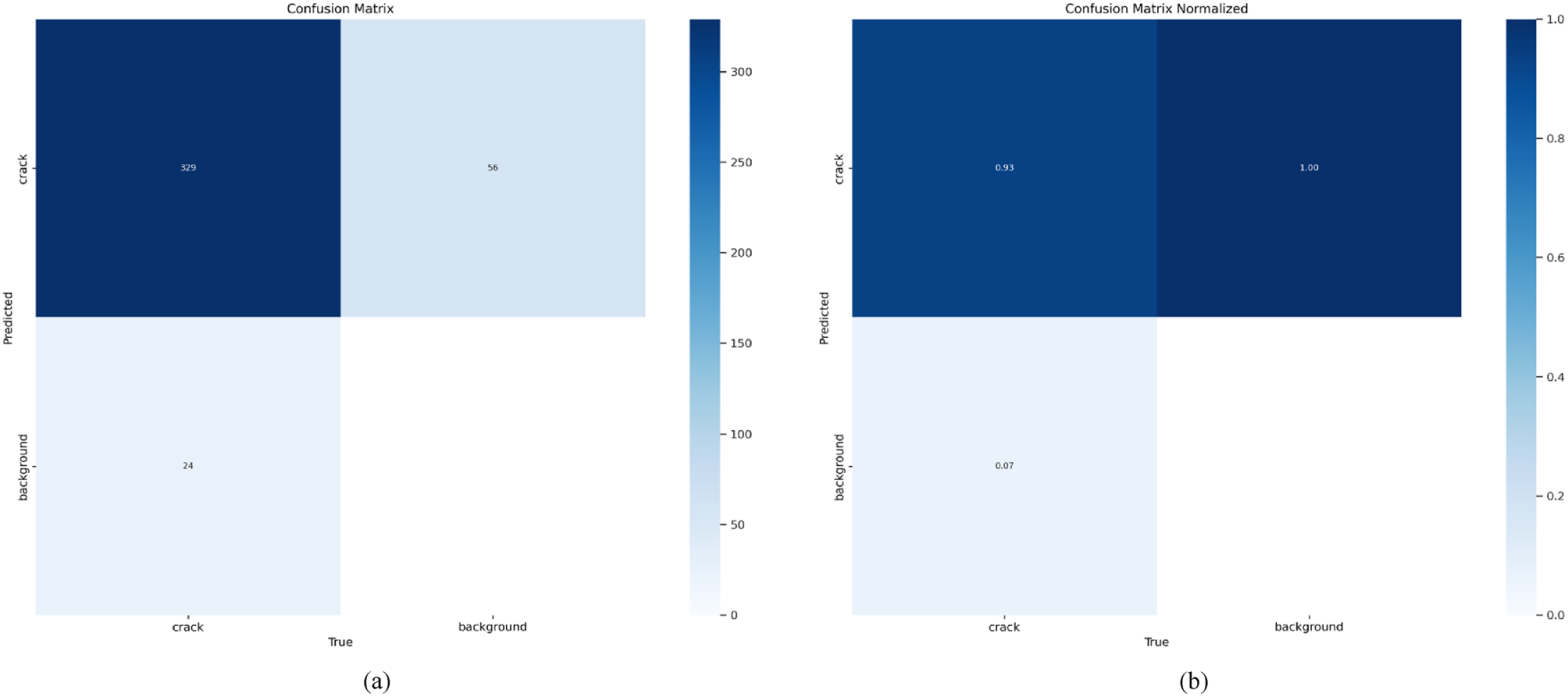
Confusion matrices of the proposed algorithm. (**a**) Standard matrix, and (**b**) normalized matrix.

The key parameters for model training are set as follows. Image size serves as a crucial determinant of input data characteristics. The input image size was fixed at 640$\times$640 pixels, as YOLO uses a fixed size to balance efficiency and speed. The batch size was set to 32, meaning 32 images were processed per parameter update. The model was trained for 2000 epochs, where one epoch represents a complete pass through the entire training dataset. The learning rate defines the step size for updating network weights during optimization. It critically influences whether and when the loss function converges to a local minimum. Therefore, selecting an appropriate learning rate is essential for effective model training. It was initialized to 0.01 to regulate weight optimization and ensure stable convergence. The auto optimizer mode was selected, which automatically adjusts optimization parameters based on real-time performance metrics during the experiment. The experiment employed stochastic gradient descent (SGD) with automated hyperparameter tuning, supplemented by an early stopping mechanism that halted training if no improvement was observed for 100 consecutive epochs.

### Evaluation metrics

After completing the model experiments, it is essential to assess the model’s ability in accurately identifying and locating objects within test images. The core of this evaluation is measuring the overlap between the predicted box and the GT box, using the Intersection over Union (IoU) metric. Typically, a detection is considered correct only if the IoU exceeds the standard threshold of 0.5.

Evaluating object detection performance primarily focuses on accuracy and speed. Prior to introducing specific metrics, we first define the confusion matrix, which summarizes classification outcomes by comparing predictions against GT. Data within the matrix forms the basis for computing all subsequent evaluation metrics. For binary classification, the matrix comprises four fundamental elements: true positives (TP), false positives (FP), true negatives (TN), and false negatives (FN). Clearly, these elements encompass all possible prediction scenarios. Figure 7 provides visual examples to clarify the evaluation metrics used in bridge crack detection. Figure 7a displays a sample image containing cracks. TP refers to images with cracks that are correctly detected, as shown in Fig. 7b. FP denotes images without cracks where cracks are falsely detected, as shown in Fig. 7c. TN refers to images without cracks correctly identified as crack-free, as shown in Fig. 7d. FN indicates images with cracks that are missed by the model, as shown in Fig. 7e. Figure 7f illustrates the calculation of Intersection over Union (IoU) for a candidate detection.

**Table 1:** Hyperparameter sensitivity analysis for the C2LSKA module.

Configuration (N, k, d)	P (%)	R (%)	$F_{1}$	mAP@50 (%)	mAP@50-95 (%)	Params	GFLOPs	FPS
(1, 7, 2)	90.0	90.9	0.904	92.9	65.1	922,221,1	21.2	434.8
(1, 11, 2)	92.9	89.5	0.912	92.9	65.0	922,323,5	21.2	384.6
(1, 11, 3)	91.4	89.8	0.906	92.9	66.7	922,323,5	21.2	434.8
**(1, 23, 2)**	**93.0**	**90.3**	**0.916**	**93.0**	**67.5**	**922,630,7**	**21.2**	**434.8**
(2, 23, 2)	93.0	90.3	0.916	93.0	67.5	922,630,7	21.2	434.8
(4, 23, 2)	93.0	90.3	0.916	93.0	67.5	922,630,7	21.2	416.7
(1, 35, 2)	91.0	90.1	0.905	93.0	66.2	922,937,9	21.2	454.5

Significant values are in bold.

This paper adopts precision (P), recall (R), mAP@50, and mAP@50-95 as evaluation criteria, with primary emphasis on mAP@50 and mAP@50-95. P reflects the proportion of true positives among all detections predicted as positive, measuring the model’s exactness. A higher precision indicates a greater ratio of TP to FP, calculated as 6:

6 $$\begin{aligned} P=\frac{TP}{TP+FP} \end{aligned}$$

R, also known as sensitivity or true positive rate (TPR), measures the proportion of actual positives correctly identified, evaluating the completeness of detection. A high recall indicates a strong ability to capture target features (e.g., structural cracks), even if it comes with more false positives. It is defined as follows 7:

7 $$\begin{aligned} R=\frac{TP}{TP+FN} \end{aligned}$$

Average precision (AP) represents the area under the precision-recall curve for a single class relative to the coordinate axes. Mean AP (mAP) is simply the average of AP values across all classes. Higher AP and mAP values typically indicate better model performance. Their formal definitions are given in Eqs. 8 and 9, respectively.

8 $$\begin{aligned} AP=\frac{1}{N}\sum ^{N}_{i=1}P_{i} \end{aligned}$$

9 $$\begin{aligned} mAP=\frac{1}{C}\sum ^{C}_{i=1}AP_{i} \end{aligned}$$

where N denotes the number of tested images, and C denotes the total number of detection categories used.

The $F_{1}$ score is the harmonic mean of precision and recall. It serves as a balanced evaluation metric by integrating both. The formula is expressed as 10:

10 $$\begin{aligned} F_{1} =\frac{2\times P\times R}{P+R} \end{aligned}$$

The processing latency in the YOLO architecture consists of three sequential stages: input preprocessing, network inference, and output postprocessing. This total latency is a critical metric for assessing model efficiency and real-time capability. Closely related is Frames Per Second (FPS), which measures throughput by counting how many images can be processed per second. FPS is widely used to evaluate vision models, where a higher value indicates faster speed and better suitability for real-time applications. It is calculated as shown in formula 11:

11 $$\begin{aligned} FPS=\frac{FrameNum}{ElapsedTime} \end{aligned}$$

where FrameNum denotes the total number of processed images, and ElapsedTime represents the total inference time consumed by the model. Giga FLOPs (GFLOPs) quantifies the theoretical peak performance of processing units like GPUs, with higher values indicating greater potential computational throughput.

## Experiment and result analysis

The experimental analysis was structured into four main phases: a hyperparameter sensitivity analysis for the C2LSKA module, an examination of the training curves, ablation studies to assess the contribution of individual components, and comparisons against mainstream detection methods.

### Hyperparameter sensitivity analysis for the C2LSKA module

To systematically investigate the influence of the C2LSKA module’s architectural hyperparameters on bridge crack detection performance, a dedicated hyperparameter sensitivity analysis was conducted. The primary objective was to identify the optimal configuration that maximizes crack detection accuracy while maintaining computational efficiency suitable for real-time applications. The analysis focused on three core parameters: the number of cascaded LSKA units (N), the kernel size of the large-kernel convolution (k), and the dilation rate (d). These parameters critically govern the module’s multi-scale receptive field and its capacity for contextual feature integration, which are essential for reliably detecting cracks of varying widths and against complex backgrounds.

**Table 2:** The results of the ablation experiment.

Model	C2LSKA	GD	SIoU	P (%)	R (%)	${F_{1}}$	mAP@50 (%)	mAP@50-95 (%)
YOLO11	$\times$	$\times$	$\times$	89.5	89.2	0.907	92.3	65.3
YOLO11_01	$\surd$	$\times$	$\times$	**93.0**$\uparrow$	90.3	0.916	93.0	67.5
YOLO11_02	$\times$	$\surd$	$\times$	90.1	89.0	0.895	92.3	66.1
YOLO11_03	$\times$	$\times$	$\surd$	92.0	91.5	0.917	93.4	66.2
YOLO11_04	$\surd$	$\surd$	$\times$	92.6	89.5	0.910	93.2	66.5
YOLO11_05	$\surd$	$\times$	$\surd$	90.8	92.3	0.915	92.9	65.6
YOLO11_06	$\times$	$\surd$	$\surd$	92.2	**92.4**$\uparrow$	**0.923**$\uparrow$	93.3	68.1
**Our method**	$\surd$	$\surd$	$\surd$	90.7	90.1	0.904	**93.5**$\uparrow$	**68.5**$\uparrow$

Significant values are in bold.

The experimental design involved a comprehensive grid search over defined parameter ranges: N $\in${1, 2, 4}, k$\in${7, 11, 23, 35}, and d$\in${2, 3}. The search commenced with an initial configuration of (N=1, k=7, d=3). All other network components and training hyperparameters were held constant across experiments to isolate the effect of the C2LSKA module’s structure. Each model variant was trained and evaluated on the bridge crack dataset, with performance assessed using P, R, $F_{1}$ mAP@50, mAP@50-95, Params, GFLOPs, and FPS. The summarized results are presented in Table 1.

As summarized in Table 1, increasing k from 7 to 23 generally improved detection performance, with the configuration (1, 23, 2) achieving the highest mAP@50–95 (67.5%) and $F_{1}$ (0.916). However, further increasing k to 35 did not yield consistent gains, indicating a potential saturation in receptive field benefit. Variations in d showed that d=2 consistently outperformed or matched d=3 across comparable settings, suggesting that moderate dilation effectively balances contextual integration without overly sparsifying feature sampling. Notably, increasing N from 1 to 4 while keeping k=23 and d=2 resulted in identical precision, recall, F1, and mAP metrics, though with a slight drop in FPS from 434.8 to 416.7, implying diminishing returns from deeper cascading under fixed kernel and dilation settings. Across all trials, the configuration (1, 23, 2) provided an optimal balance, attaining 93.0% precision, 90.3% recall, 0.916 $F_{1}$, 93.0% mAP@50, and 67.5% mAP@50–95, while maintaining competitive efficiency (21.2 GFLOPs, 434.8 FPS). These results validate that a carefully tuned C2LSKA module—with moderate kernel size, appropriate dilation, and shallow cascade depth—can achieve high detection accuracy without compromising runtime performance, making it well-suited for real-world bridge inspection scenarios.

### Analysis of training results graph

The training dynamics and convergence behavior of the proposed model were systematically monitored by tracking key loss components and performance metrics throughout the training process. The model’s loss function consists of three main parts: the bounding box loss (box_loss), the classification loss (cls_loss), and the DFL loss (dfl_loss). The box_loss measures how well the predicted bounding boxes match the actual ones. Reducing this loss improves the model’s ability to locate objects accurately. The cls_loss evaluates errors in predicting object categories. Lowering this loss helps the model better distinguish between different classes. The dfl_loss captures differences in angular orientation. Minimizing this loss leads to more precise rotation predictions and enhances the model’s ability to capture fine-grained features.

Figure 8a, b demonstrate the training and test loss curves of YOLO11 and the proposed algorithm, respectively. All primary loss functions exhibited a stable and monotonic decline throughout the training process, indicating effective optimization. On the training set, the bounding box regression, classification, and distribution focal Loss demonstrated coordinated convergence. Starting from initial values of approximately 3.4, they decreased consistently, reaching a plateau near 0.1 by the final epoch. This synchronized reduction suggests balanced learning across localization and classification tasks. A corresponding trend was observed on the validation set. The validation bounding box and classification loss decreased sharply from initial values of 3.0 and 3.0, respectively, converging to near-zero values after 80 epochs. Similarly, the validation DFL loss declined from 4.0 to 0.02. The rapid and parallel decay of these validation losses, coupled with the absence of subsequent divergence, indicates that the model generalized effectively without overfitting to the training data.

Alongside loss convergence, key detection metrics were tracked to evaluate practical performance. Figure 8c–f depict the recall, precision, mAP@50, and mAP@50-95 curves over 100 training epochs. All metrics rise sharply at first and then gradually stabilize, converging around epoch 200. This trend confirms the steady performance improvement and enhanced crack detection capability throughout training.

**Table 3:** Ablation experiment.

Model	C2LSKA	GD	SIoU	Layers	Params	GFLOPs	FPS
YOLO11	$\times$	$\times$	$\times$	100	9,413,187	21.3	**434.8**
YOLO11_01	$\surd$	$\times$	$\times$	108	**9,226,307**	21.2	**434.8**
YOLO11_02	$\times$	$\surd$	$\times$	239	13,799,651	**28.8**	217.4
YOLO11_03	$\times$	$\times$	$\surd$	100	9,413,187	21.3	400.0
YOLO11_04	$\surd$	$\surd$	$\times$	247	13,612,771	28.7	212.8
YOLO11_05	$\surd$	$\times$	$\surd$	108	**9,226,307**	21.2	384.6
YOLO11_06	$\times$	$\surd$	$\surd$	239	13,799,651	**28.8**	232.6
**Our method**	$\surd$	$\surd$	$\surd$	247	13,612,771	28.7	222.2

Significant values are in bold.

Figure 9 compares the mAP@50 precision-recall curves of YOLO11 and our proposed algorithm, illustrating the characteristic trade-off where higher precision reduces recall. Our method’s curve, positioned closer to the top-right corner, achieves a superior mAP@50 of 93.5%, marking a 1.2% improvement over YOLO11.

To complement quantitative metrics, we calculated additional evaluation curves for a comprehensive visual analysis of model performance. These include the $F_{1}$-confidence, precision-confidence (PC), and recall-confidence (RC) curves, shown in Fig. 10. They provide visual insights into detection accuracy: the $F_{1}$ curve reveals the precision-recall trade-off across confidence levels; the PC curve shows precision rising with higher confidence; and the RC curve indicates stable recall performance (a large AUC suggests few missed detections). Notably, the PC curve peaks at an $F_{1}$ score of 0.89, corresponding to a confidence threshold of 0.320. Figure 11 expounds the confusion matrix of our algorithm, with columns representing GT classes and rows indicating predicted labels. The strong diagonal pattern confirms an overall accuracy of 93%, quantitatively validates the model’s strong discriminative capability in identifying structural defects.

### Ablation experiments

To validate the contribution of each proposed component (C2LSKA, GD, and SIoU), we conduct comprehensive ablation experiments on both detection performance (Table 2) and computational complexity (Table 3). Each component was added incrementally while keeping the overall architecture consistent.

**Table 4:** Comparison experiment.

Method	P (%)	R (%)	$F_{1}$	mAP@50 (%)	mAP@50-95 (%)	Params	GFLOPs	FPS
SSD	**93.2**$\uparrow$	77.9	0.849	90.6	-	28.8(M)	250.14	-
Faster R-CNN	77.9	**92.6**$\uparrow$	0.846	91.2	-	23.75(M)	30.43	-
YOLOv5	89.2	90.1	0.896	92.0	60.3	781,400,3	18.7	**500.0**
YOLOv8	91.1	88.4	0.897	92.9	62.6	982,805,1	23.3	434.8
YOLO11	89.5	92.0	**0.907**$\uparrow$	92.3	65.3	941,318,7	21.3	434.8
**Our method**	90.7	90.1	0.904	**93.5**$\uparrow$	**68.5**$\uparrow$	136,127,71	**28.7**	222.2

Significant values are in bold.

The baseline YOLO11 achieves 89.5% precision, 89.2% recall, 0.907 $F_{1}$, 92.3% mAP@50, and 65.3% mAP@50-95, with 9,413,187 parameters, 21.3 GFLOPs, and 434.8 FPS. Each modification contributed to a performance gain,increasing mAP@50-95 by 2.2% Incorporating the GD mechanism further increased mAP@50-95 by 0.8%. Subsequently, integrating the SIoU loss contributed an additional 0.9%. The full model integrating all three improvements achieved an mAP@50-95 of 68.5%, a 3.2% gain over the baseline. Table 2 details this stepwise optimization.

**Fig. 12:**
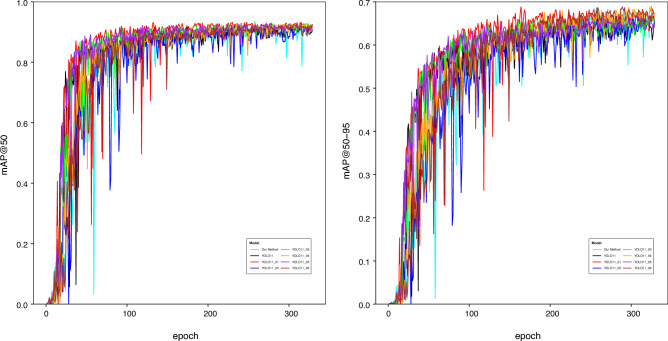
Accuracy curves (left: mAP@50, right: mAP@50-95) of ablation study.

Ablation experiments demonstrate that incorporating the C2LSKA module (YOLO11_01) significantly boosts YOLO11’s detection performance. Precision rises to 93.0%, recall to 90.3%, and the $F_{1}$ score to 0.916, with corresponding gains in both mAP@50 and mAP@50-95. While the original C2PSA module in YOLO11 enables effective multi-scale feature fusion through varied max-pooling kernels, it does so at a substantial computational cost. In contrast, the proposed C2LSKA module preserves this multi-scale capability to capture rich contextual information, but replaces the heavy components with LSKA attention. By decomposing large-kernel convolutions, LSKA efficiently captures multi-dimensional regional features to resolve cross-dimensional relationships, thereby enhancing detection accuracy while significantly reducing computational overhead. To prevent feature fragmentation from multidimensional processing, LSKA employs a final fusion operation to ensure information continuity. As a plug-and-play component, C2LSKA offers task-specific flexibility (e.g., parameter tuning for occluded or small-object scenarios), which further improves fine-grained feature representation and overall model generalization.

The synergistic integration of the GD mechanism (YOLO11_02) improves YOLO11’s mAP@50-95 through its dual-path (Low-GD and High-GD) feature integration across backbone levels, which enhances feature representation. Precision (90.1%) and recall (89.0%) remain close to baseline, leading to a slightly lower $F_{1}$ (0.895). By combining convolutions with self-attention, the GD mechanism leverages both local pattern extraction and global dependency modeling, mitigating the spatial limitations of single-branch attention. To prevent information loss during cross-layer interaction, the GD mechanism incorporates FAM, IFM, and an Inject module. FAM aligns cross-level features, IFM facilitates multi-source feature mixing, and Inject enhances global representations. This integrated framework significantly improves the extraction of critical details and contextual understanding. Furthermore, the GD mechanism performs dual nonlinear channel recalibration, compressing redundant information while amplifying task-relevant features. This discriminative feature refinement capability provides a superior foundation for both classification and localization tasks. Despite the increased complexity, mAP@50-95 improves to 66.1%, indicating that dual-path feature fusion enhances multi-scale representation at a measurable computational cost.

Replacing the default loss with SIoU (YOLO11_03) yields 92.0% precision, 91.5% recall, 0.917 $F_{1}$, 93.4% mAP@50, and 66.2% mAP@50-95 without altering parameters or GFLOPs. This significant gain validates the efficiency and synergistic potential of these components. The SIoU loss specifically advances localization by explicitly modeling the angle, distance, and shape of targets, overcoming the scale sensitivity of traditional IoU. Its design applies a scale-invariant principle that normalizes dimensions to relative ratios during IoU calculation, eliminating bias from absolute target size. A dynamic weighting mechanism also auto-adjusts penalties for positional and shape deviations based on target scale, preventing small targets from being excessively penalized. By integrating these geometric constraints, SIoU boosts accuracy in dense scenes and on targets with extreme aspect ratios, such as cracks.

**Fig. 13:**
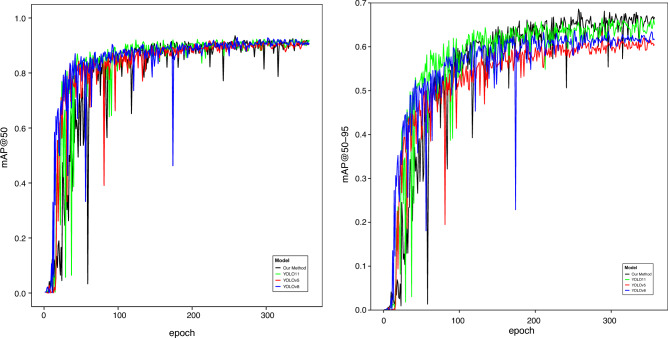
Comparison of mAP (left: mAP@50, right: mAP@50-95) curves for YOLO-series algorithms.

When C2LSKA and GD are jointly applied (YOLO11_04), the model reaches 247 layers, 13.61M parameters, and 28.7 GFLOPs, closely matching the footprint of GD alone. Performance (93.2% mAP@50, 66.5% mAP@50-95) surpasses either module individually, demonstrating synergy between efficient attention and multi-scale fusion. The recall of GD and SIoU is notably high, but the precision drops compared to C2LSKA alone, suggesting that the joint application may slightly over-sensitize the detector. The combination of GD and SIoU (YOLO11_06) achieves the highest $F_{1}$ score (0.923) and a substantial mAP@50-95 of 68.1%, highlighting the complementary roles of feature integration and advanced loss design. Our method attains the highest mAP@50 (93.5%) and mAP@50-95 (68.5%), outperforming all ablated counterparts. Although precision (90.7%) and $F_{1}$ (0.904) are slightly lower than some individual variants, the consistent gains under stricter metrics demonstrate superior localization robustness.

**Table 5:** Results of bridge crack detection on simulated data.

Conditions	Models	Params (M)	FLOPs (G)	P (%)	R (%)	mAP@50 (%)	mAP@50-95 (%)
Original image	YOLO11	9.428	21.548	89.3	92.1	92.3	65.2
	Ours	13.626	28.954	91.1	89.8	93.5	68.6
Over-exposure	YOLO11	9.428	21.548	90.7	91.4	92.1	65.8
	Ours	13.626	28.954	89.3	87.9	91.2	66.1
Under-exposure	YOLO11	9.428	21.548	88.6	92.1	93.0	66.2
	Ours	13.626	28.954	90.5	91.2	93.1	67.8
Gaussian noise	YOLO11	9.428	21.548	89.2	84.6	88.2	57.0
	Ours	13.626	28.954	85.6	84.1	84.7	59.2
Salt-and-pepper noise	YOLO11	9.428	21.548	88.9	91.8	91.1	68.2
	Ours	13.626	28.954	90.1	89.8	92.5	68.0
Speckle	YOLO11	9.428	21.548	90.6	89.8	91.6	65.1
	Ours	13.626	28.954	91.2	89.0	93.3	67.6
Random colored line artifacts	YOLO11	9.428	21.548	67.7	64.6	59.7	34.1
	Ours	13.626	28.954	67.9	67.1	64.5	40.3

Regarding model performance, the baseline YOLO11 achieved 434.8 FPS. C2LSKA adds only 8 layers, while maintaining identical inference speed (434.8 FPS). This confirms the exceptional efficiency of the proposed kernel decomposition strategy. GD substantially increases parameters and GFLOPs. This reflects the inherent computational cost of dual-path multi-scale feature integration. SIoU introduces no change in layers, parameters, or GFLOPs. The only observable effect is a marginal FPS reduction, attributable to the additional geometric calculations in the loss function. The experimental results obtained for the YOLO11_04 variant demonstrate that the C2LSKA module integrates seamlessly with the GD mechanism, incurring no additional computational cost beyond that intrinsic to GD itself. Furthermore, the combination of C2LSKA and SIoU reveals that SIoU introduces negligible overhead when paired with C2LSKA. Similarly, the GD+SIoU variant confirms that SIoU imposes no measurable architectural burden and may even facilitate marginal throughput gains, likely attributable to improved convergence behavior.

**Fig. 14:**
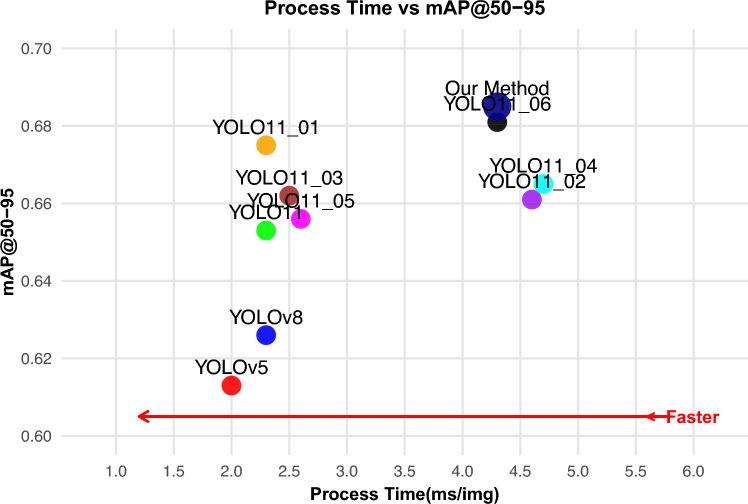
The trade-off between detection accuracy (mAP@50-95) and speed (processing time) for different YOLO architectures and their ablated variants.

The ablation study elucidates the distinct roles and synergistic effects of the proposed modules. C2LSKA delivers consistent accuracy gains with zero inference speed penalty and even reduces parameter count, making it a highly efficient attention enhancer suitable for resource-sensitive applications. GD provides substantial improvements in multi-scale representation and localization accuracy–particularly under stricter IoU thresholds–but at the expense of increased depth, parameters, and latency. This trade-off is justified by the corresponding performance lift and remains acceptable for real-time deployment (217–232 FPS). SIoU refines bounding box regression, boosting precision and recall with negligible computational overhead and only a minor FPS reduction, demonstrating its practicality as a drop-in loss function. The full model attains the best overall detection accuracy (93.5% mAP@50, 68.5% mAP@50-95), validating that all three components work synergistically. Although the full model does not maximize every individual metric (e.g., precision, $F_{1}$), the consistent elevation under strict IoU thresholds confirms its superior localization capability and robustness. From an efficiency perspective, the full model remains suitable for real-time deployment (222.2 FPS) with a moderate parameter increase relative to the baseline. This favorable balance between accuracy and computational cost supports its practical applicability in resource-constrained bridge inspection scenarios.

**Fig. 15:**
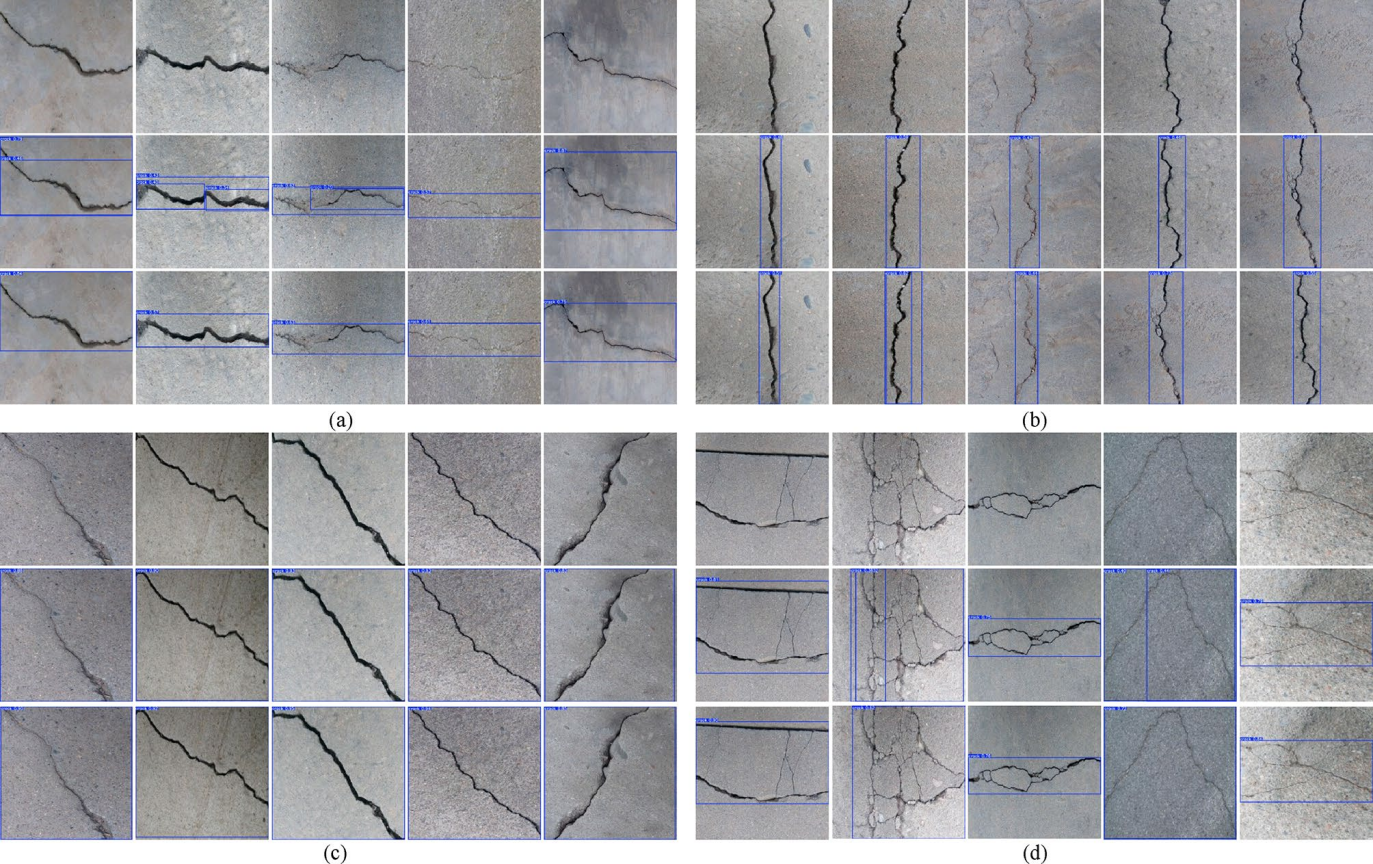
Performance metric comparison on the crack detection dataset. (**a**) Transverse crack detection, (**b**) longitudinal crack detection, (**c**) diagonal crack detection, and (**d**) detection efficacy for complex morphologies.

Figure 12 employs line graphs to illustrate the mAP@50 and mAP@50-95 convergence trends from the ablation study. The curves YOLO11_01, YOLO11_02, YOLO11_03, YOLO11_04, YOLO11_05 and YOLO11_06 correspond to adding the C2LSKA module, GD mechanism, SIoU loss, C2LSKA+GD, C2LSKA+SIoU, and GD+SIoU, respectively. The proposed architecture shows faster convergence and sustained superior performance in later training.

### Comparison with the baselines

We evaluated our bridge crack detection method by comparing it with mainstream object detection approaches. All comparisons used the same dataset, training environment, hardware, hyperparameters, and data augmentation to guarantee reliability. The following benchmark algorithms were used for comparison:

- SSD^28^: SSD is a foundational, end-to-end single-stage object detection framework. Its design achieves an effective balance between accuracy and speed by using multi-scale feature maps and predefined default boxes. A key innovation of SSD is the removal of the region proposal stage, which significantly improves inference efficiency;
- Faster R-CNN^26^: Faster R-CNN introduces a trainable two-stage detection framework. It shares convolutional features between the Region Proposal Network (RPN) and the detection network, eliminating the computational overhead of earlier two-stage models like R-CNN and Fast R-CNN. This allows it to achieve higher accuracy and faster inference;
- YOLOv5^40^: YOLOv5 introduces architectural refinements over YOLOv3/v4 through modular design and enhanced training pipelines. This industry-oriented framework maintains real-time efficiency while significantly boosting small-object detection accuracy;
- YOLOv8^40^: YOLOv8 (Ultralytics, 2023) employs an anchor-free architecture within a unified multi-task framework. Its core innovations include a scalable backbone and task-decoupled heads, achieving state-of-the-art accuracy in detection, segmentation, and pose estimation with efficient inference;
- YOLO11^40^: YOLO11 integrates transformers with CNNs into a lightweight dual-branch architecture. It retains the efficiency of single-stage detectors while effectively addressing small-object detection, long-tail distribution adaptation, and multimodal feature fusion.

Table 4 presents a quantitative comparison between our proposed method and several mainstream object detection architectures on the bridge crack detection task. The results demonstrate that our method achieves the highest detection accuracy among all compared approaches, with a mAP@50 of 93.5% and a mAP@50-95 of 68.5%—surpassing the baseline YOLO11 by +1.2% and +3.2%, respectively. Although YOLO11 achieves the highest $F_{1}$ score (0.907) and competitive inference speed (434.8 FPS), our method attains superior overall localization performance, particularly under stricter IoU thresholds (mAP@50-95), indicating better bounding box precision and scale robustness. Compared with YOLOv5, YOLOv8, and YOLO11, our model improves mAP@50-95 by 8.2%, 5.9%, and 3.2%, respectively, confirming the effectiveness of the proposed C2LSKA module and GD mechanism in enhancing multi-scale feature representation and crack discrimination. In terms of model complexity, our method introduces 13,612,771 parameters and 28.7 GFLOPs, which is higher than YOLO11, and with a reduced inference speed of 222.2 FPS. This trade-off reflects the additional computational cost introduced by the dual-path feature integration. Nevertheless, the method remains suitable for real-time deployment and offers a favorable balance between accuracy and efficiency. Overall, the comparative analysis validates that our method achieves state-of-the-art performance on the bridge crack detection benchmark, with clear advantages in both mAP@50 and mAP@50-95.

**Fig. 16:**
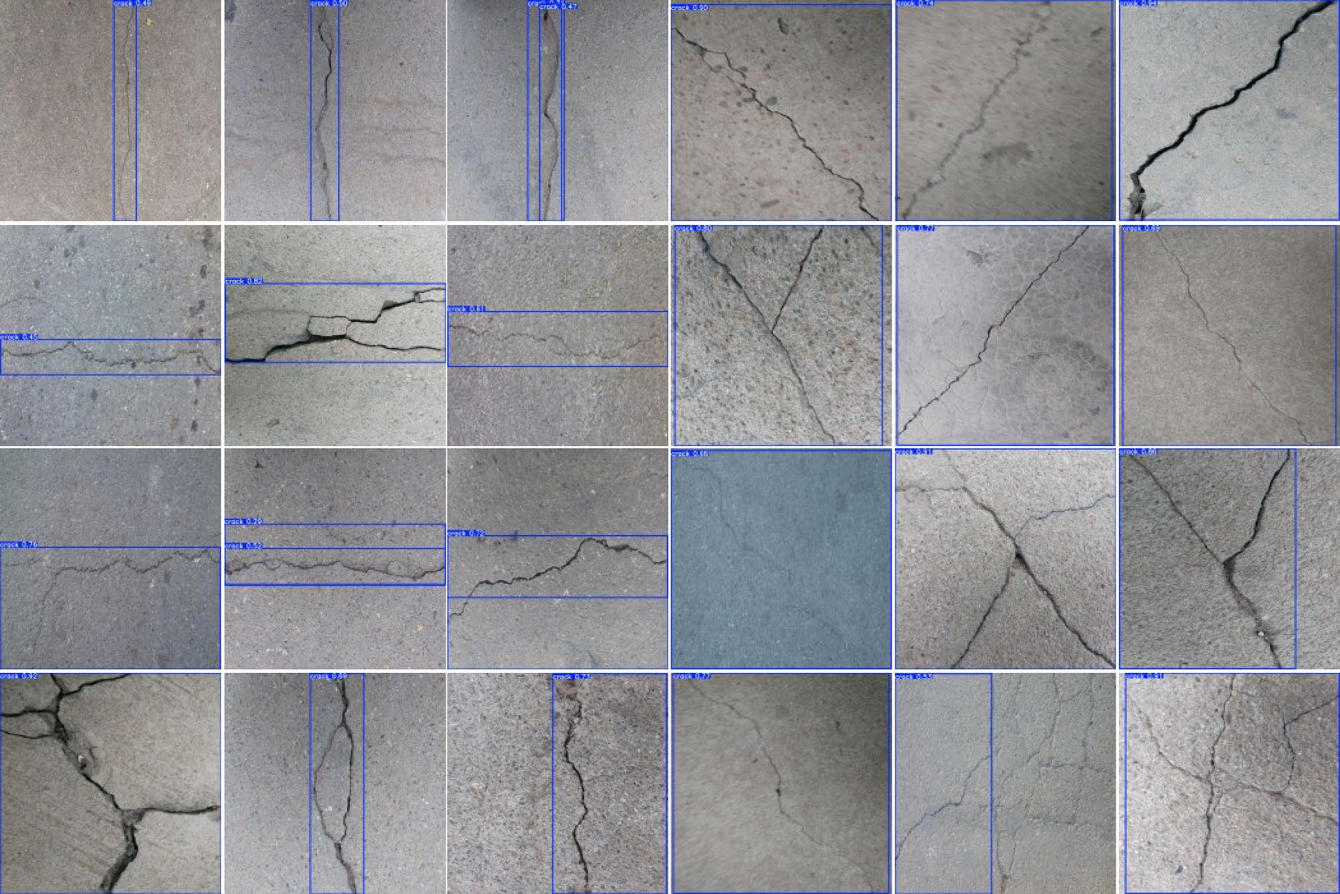
Representative detection results.

The line graphs in Fig. 13 further present the mAP@50 and mAP@50-95 curves of our algorithm compared to other YOLO models. As shown, the proposed algorithm exhibits lower and more fluctuating mAP values during the initial training phase (with smaller epochs). After around 100 epochs, it stabilizes and converges quickly, eventually maintaining a stable, leading performance. The scatter plot in Fig. 14 illustrates the speed-accuracy trade-off between processing time and mAP@50-95. Although our algorithm achieves higher mAP than other methods, its inference speed is not the fastest. Nonetheless, it offers a practical balance between real-time performance and detection accuracy.

In YOLO-based evaluation, the confidence score (ranging from 0 to 1) quantifies the certainty of a detection. Figure 15 presents a visual comparative analysis of crack detection results from YOLOv5, YOLOv8, YOLO11, and our proposed method. The first column shows original images with diverse crack types, while columns two to five display the respective detection results. Specifically, subfigure (a) compares transverse crack detection, (b) contrasts longitudinal cracks, and (c) evaluates performance on complex morphologies (e.g., reticulated and diagonal cracks). The results demonstrate our algorithm’s superior robustness. It effectively identifies multi-scale, irregular cracks with significant morphological variations, such as branching and discontinuous patterns.

Figure 16 shows representative detection results of our algorithm on the test set. As shown in the figure, the model accurately identifies various types, including transverse, longitudinal, and reticulated cracks, demonstrating high precision and robustness even for thin, low-contrast cracks in challenging conditions. These results validate the generalization capability enabled by our multi-scale feature fusion mechanism.

**Fig. 17:**
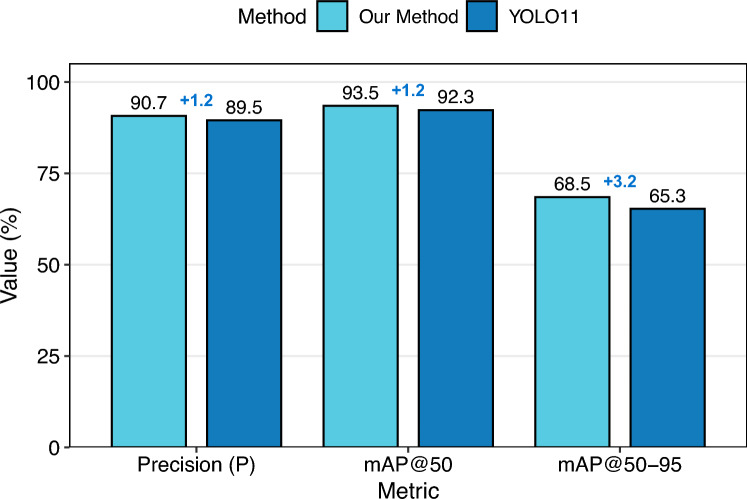
Comparison of performance metrics on the crack detection dataset.

In summary, our method achieves substantial improvements in bridge crack detection accuracy under constrained model complexity, with absolute gains of 1.2% in Precision, 1.2% in mAP@50, and 3.2% in mAP@50-95 compared to the baseline, as detailed in Fig. 17. Unlike traditional two-stage detectors such as Faster R-CNN, which are limited by inherent trade-offs between precision and efficiency, our approach effectively solves two key challenges: discriminating fine cracks and suppressing crack-like noise artifacts, achieving a superior balance of accuracy, robustness, and speed.

### Robustness analysis

A primary challenge in deploying vision-based crack detection models in the field is their performance degradation under suboptimal imaging conditions, such as sensor noise, adverse weather, partial occlusions, and the presence of confounding linear structures (e.g., construction joints, scratches). To proactively assess and validate the robustness of the proposed CLGDS-Net against such real-world perturbations, a dedicated experiment was conducted using a suite of targeted data augmentation techniques on the test set. The objective was to simulate these challenging conditions during inference to evaluate the model’s resilience and generalization capability beyond its training distribution, thereby providing a more realistic estimate of its operational reliability.

To simulate challenging real-world conditions, the original test images were augmented with the following common yet disruptive perturbations: varying illumination to mimic different lighting environments; Gaussian noise to approximate electronic sensor noise or low-light grain; salt-and-pepper noise to emulate surface impurities such as dust or raindrops; speckle noise to represent granular interference common in active imaging systems; randomly colored lines of varying thickness and orientation to imitate non–crack linear features that may cause false positives. The proposed model was benchmarked against YOLO11 across multiple metrics, including Params, FLOPs, P, R, and mAP.

**Fig. 18:**
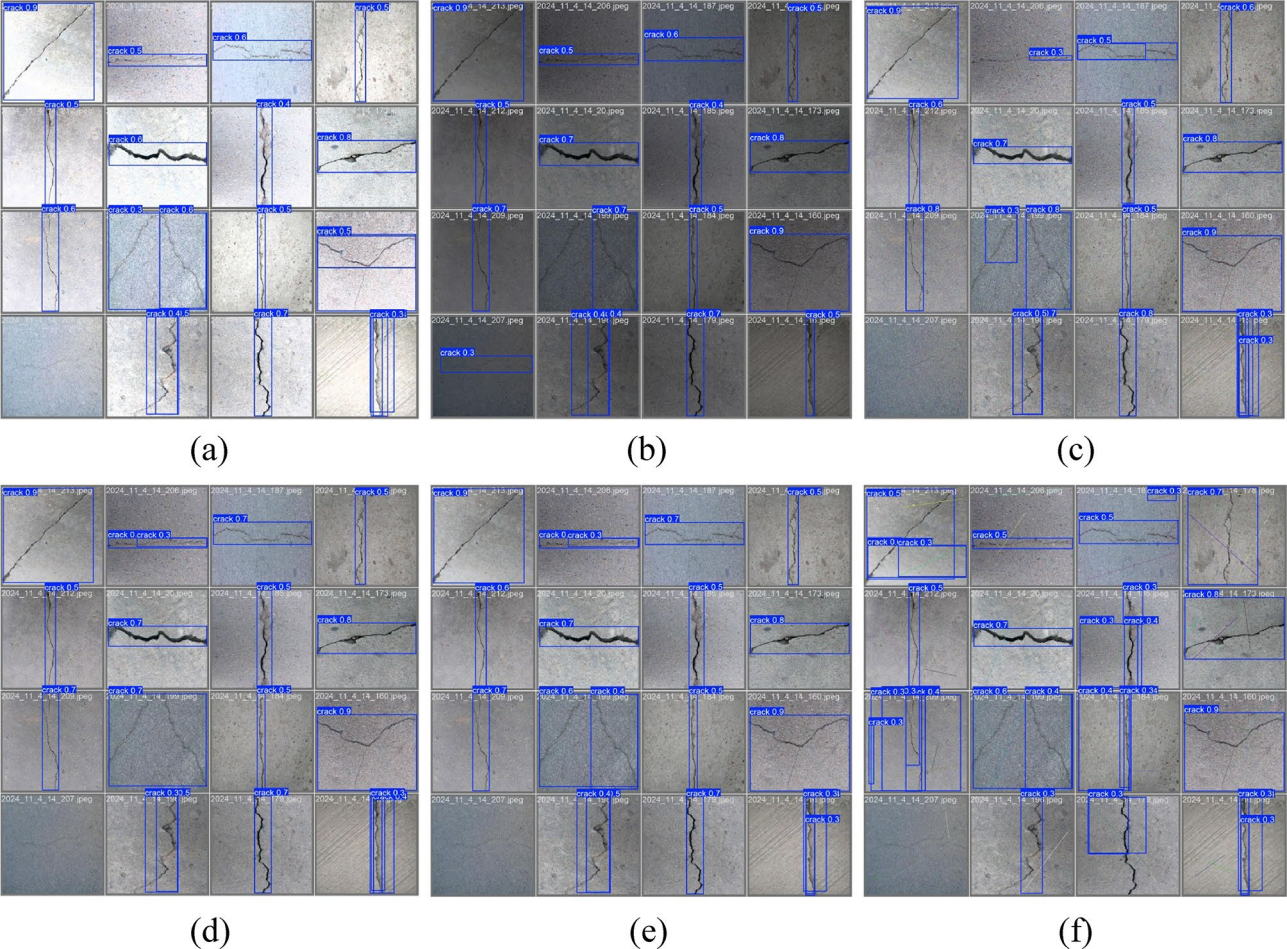
Visualized detection results of bridge cracks under simulated degraded conditions. (**a**) Over‑exposure, (**b**) under-exposure, (**c**) Gaussian noise, (**d**) salt-and-pepper noise, (**e**) speckle noise, and (**f**) randomly colored lines.

The detection performance under these augmented conditions is summarized in Table  5 and Fig. 18. As reported in the table, the proposed model shows distinct behaviors under varying illumination: under over-exposure, its precision drops slightly more than YOLO11, resulting in a moderately lower mAP@50 (-0.9%). In under-exposed scenes, however, it maintains more balanced performance and surpasses YOLO11 in mAP@50 (+0.2%). Regarding noise robustness, both models decline notably under Gaussian noise, with the proposed model exhibiting a larger precision decrease (85.6%) yet retaining a slightly higher mAP@50-95 (+2.2%), indicating somewhat better localization consistency. For discrete noise types–salt-and-pepper and speckle noise–the proposed model consistently outperforms YOLO11 across all mAP metrics, confirming its stronger resilience to such pixel-level corruptions. This confirms that the proposed architecture provides a robust crack detection solution under diverse degraded imaging conditions. It demonstrates superior resilience to speckle noise, salt-and-pepper noise, and structural line artifacts compared to the YOLO11 baseline, as visually supported by the detection consistency shown in Fig. 18. However, it remains sensitive to Gaussian noise and over-exposure.

## Discussion

This work presents CLGDS-Net, an improved bridge crack detection algorithm based on YOLO11. While comparative experiments confirm its precision advantage over other models in the YOLO series (Tables 2 and 3), our method, as shown in Table 3, still slightly lags behind the SSD framework in certain accuracy metrics and occasionally generates false positives or negatives. These limitations primarily stem from two factors: (1) dataset scale constraints: reliance on public datasets with limited samples and insufficient coverage of diverse crack morphologies and background environments; and (2) feature representation limitations: although the introduced GD mechanism enhances accuracy over YOLO series models (Tables 2 and 3), it increases computational complexity. Consequently, although the proposed method demonstrates superior performance on available benchmarks, its generalization capability in real-world environments–where material texture, lighting conditions, surface degradation (e.g., seepage stains in tunnels, weathering on dams), and crack types differ substantially—remains to be fully verified. Future work will therefore focus on: (1) curating or constructing larger, more diverse multi-scene datasets (e.g., tunnels, dams) to improve model robustness and reliability; and (2) optimizing feature extraction and fusion techniques to enhance crack detection accuracy. However, it is noted that these improvements often come at the cost of increased model complexity, which can adversely affect real-time performance and deployment feasibility. Subsequent research should, therefore, also explore lightweight model design, compression techniques, and computational resource optimization for efficient embedded deployment. In summary, further advancements in this domain will target synergistic progress in data augmentation, architectural refinement, feature learning enhancement, and hardware-aware optimization.

## Conclusions

To address the difficulty of detecting bridge cracks due to their irregular and multi-scale nature, this paper proposes a new detection network. It integrates three key components: the C2LSKA module, the GD mechanism, and the SIoU loss. The C2LSKA module employs large-kernel depthwise separable convolutions combined with a sub-pixel attention mechanism to better capture fine-grained crack features. The GD mechanism strengthens crack feature representation by building cross-level connections through two pathways, thereby helping to prevent feature loss. The SIoU loss improves the detection of irregular cracks by adding an angular penalty and using dynamic weighting. Trained and tested on a public bridge crack dataset, our method achieved a precision of 90.7%, recall of 90.1%, an $F_{1}$ score of 0.904, and an mAP@50 of 93.5%. These results show that our network performs well in clearly segmenting crack boundaries and capturing local details. It is especially effective at detecting slender micro-cracks and crack tip branches.

Experimental results demonstrate the robust generalization and detection capability of our method. The main conclusions are: (1) Both local detail and global semantic features are critical: To address the limitations of existing detection methods that rely on single-dimensional feature extraction, we propose the novel C2LSKA module. It mainly combines the advantages of C2PSA and LSKA, which can not only preserve multi-scale processing capability of C2PSA, but also employ vertically and horizontally sensitive convolutional kernels to suppress background noise interference. This significantly improves the detection of slender cracks. (2) Effective feature fusion significantly enhances representation discriminability. The introduced GD mechanism enables multi-level interaction via its Low-GD and High-GD pathways. Integrated into the YOLO11 neck, it improves mAP@50-95 by 3.2%, validating its efficacy in refining multi-scale crack features. (3) Class imbalance and crack irregularity pose key challenges. These factors induce network localization bias, compromising detection accuracy. Our method employs the SIoU loss, whose adaptive weighting mechanism dynamically balances foreground-background contributions. This reduces deviations in bounding box IoU predictions and effectively mitigates the regression bias associated with class imbalance.

However, this study acknowledges several key limitations. Detection performance declines under severe background interference, resulting in missed detections. This issue is particularly pronounced for certain crack types, such as those with extremely low contrast or complex backgrounds. Future work will focus on addressing these challenges: (1) Embedding prior knowledge. Future studies should incorporate crack geometric constraints (e.g., curvature continuity, branch angles) into the model. This would create physics-aware modules to improve the detection of slender cracks. (2) Improving cross-domain robustness. We need to develop a generalized detection framework for diverse infrastructure, such as tunnel linings under seepage or dam surfaces undergoing freeze-thaw cycles. This framework must address domain-specific complexities to ensure robust performance. (3) Multidimensional crack parameter quantification. The synergistic integration of depth sensing technologies will enable concurrent measurement of critical crack dimensions (e.g., depth, width), meeting stringent engineering accuracy standards.

## Data Availability

The datasets used and/or analysed during the current study available from the corresponding author on reasonable request.
